# Axiomatic Natural Philosophy and the Emergence of Biology as a Science

**DOI:** 10.1007/s10739-020-09609-2

**Published:** 2020-06-16

**Authors:** Hein van den Berg, Boris Demarest

**Affiliations:** 1 Department of Philosophy, Institute for Logic, Language and Computation, University of Amsterdam, Oude Turfmarkt 145, 1012 GC Amsterdam, The Netherlands; 2 Philosophisches Seminar, Heidelberg University, Schulgasse 6, 69117 Heidelberg, Germany

**Keywords:** Axiomatic ideal of science, Classical model of science, Axiomatic biology, Christian Wolff, Caspar Friedrich Wolff, Gottfried Reinhold Treviranus

## Abstract

Ernst Mayr argued that the emergence of biology as a special science in the early nineteenth century was possible due to the demise of the mathematical model of science and its insistence on demonstrative knowledge. More recently, John Zammito has claimed that the rise of biology as a special science was due to a distinctive experimental, anti-metaphysical, anti-mathematical, and anti-rationalist strand of thought coming from outside of Germany. In this paper we argue that this narrative neglects the important role played by the mathematical and axiomatic model of science in the emergence of biology as a special science. We show that several major actors involved in the emergence of biology as a science in Germany were working with an axiomatic conception of science that goes back at least to Aristotle and was popular in mid-eighteenth-century German academic circles due to its endorsement by Christian Wolff. More specifically, we show that at least two major contributors to the emergence of biology in Germany—Caspar Friedrich Wolff and Gottfried Reinhold Treviranus—sought to provide a conception of the new science of life that satisfies the criteria of a traditional axiomatic ideal of science. Both C.F. Wolff and Treviranus took over strong commitments to the axiomatic model of science from major philosophers of their time, Christian Wolff and Friedrich Wilhelm Joseph Schelling, respectively. The ideal of biology as an axiomatic science with specific biological fundamental concepts and principles thus played a role in the emergence of biology as a special science.

## Introduction

The emergence of the science of biology in France and Germany in the late-eighteenth and early-nineteenth centuries continues to attract attention from historians and philosophers of the life sciences. The fact that, compared to the physical sciences, the idea of a distinct science of life emerged so late in our history raises vexing questions. Why did people in the eighteenth century begin to regard the domain of “life” as something amenable to proper scientific inquiry, such that there could be a true “science” of “life”? And why did they begin to regard “life” as a distinct domain of phenomena that can be the object of a special science? Before the emergence of biology as an autonomous science, biological phenomena were, of course, an object of scientific study; however, in the early modern period such studies were often not considered to yield *proper* science. This raises the question concerning what constitutes a “proper science” and in what ways biological phenomena came to be seen as appropriate objects of proper science. The emerging consensus (Zammito 2018) seems to be that what changed in the course of the eighteenth century is that philosophers and scientists developed causal-historical accounts of biological form (see Sloan 1979, 1995; Bach 2001, p. 105). Such causal-historical accounts are different from previous accounts in two ways. On the one hand, they are truly explanatory: by referring to powers, laws, and principles that account for specific organic forms, they explain what, on previous accounts, could not or should not be explained (see, for instance, Richards 2002, pp. 241–245, on the role of Carl Friedrich Kielmeyer). On the other hand, such accounts were more thoroughly naturalist: they explained the specificity of organic forms by referring to the historical processes in nature that gave rise to these forms (see, for example, de Jager 1991; Richards 2002; Zammito 2018).

An important historical and philosophical question is why such accounts were suddenly developed, especially in late eighteenth-century Germany. In his *Growth of Biological Thought*, Ernst Mayr claimed that the emergence of biology as a science was possible because of the gradual decline of the mathematical model for science, with its insistence on demonstrative knowledge (1982, pp. 40–41). He suggested that the emergence of biology was due in part to an aspect of Georges Buffon’s thought that has been called “anti-mathematicism” (Demeter and Schliesser 2019; Wolfe 2019), which influenced Johann Gottfried von Herder (1744–1803) and even Immanuel Kant (1724–1804) and through them the *Naturphilosophen*. John Zammito’s recent and excellent historical work *The Gestation of German Biology*, presents a different account of the emergence of biology. It gives center stage to the “experimental Newtonianism” of Albrecht von Haller (1708–1777), who had an “impact on emergent German life science … precisely because by training and disposition Haller was not steeped in what some historians of science insist upon viewing as injurious German ‘metaphysical’ impulses” (Zammito 2018, p. 70). Although these readings are different, they both stress that the formulation of the biological principles that allowed for a causal-historical account of biological form was due to a distinctive experimental, anti-mathematical, and anti-rationalist strand of thought.

In this paper, we argue that this narrative neglects the role the axiomatic model of science played in the emergence of biology as a special science. We show that several major actors involved in the emergence of biology as a science in Germany were working with an axiomatic conception of science that goes back at least to Aristotle and was popular in mid-eighteenth-century German academic circles due to its endorsement by Christian Wolff. This axiomatic conception of science has been formulated in a precise manner by Willem de Jong and Arianna Betti, who articulated the so-called classical model of science (de Jong and Betti 2010).

The classical model of science is an interpretative framework that codifies an influential axiomatic ideal of science accepted by many past philosophers and scientists (van den Berg et al. 2018; de Jong and Betti 2010). The classical model refines previous systematizations of the Aristotelian ideal of science provided by Scholz (1975 [1930], p. 52) and Beth (1950, pp. 27–28, 1965, pp. 31–32). According to de Jong and Betti (2010, p. 187), paradigmatic expositions of the classical model are in Aristotle’s *Analytica posteriora,* book 1; the so-called *Logic of Port Royal* (1662) written by Antoine Arnauld and Pierre Nicole; and Bernard Bolzano’s *Wissenschaftslehre* (1837). The classical model has been historically validated in a number of historical studies and applied as a fruitful historical tool to study axiomatic ideals of science, among others, those of Immanuel Kant (see, for example, Betti 2010; de Jong 2001, 2010; van den Berg 2011, 2014). We will use the classical model as an interpretative tool to interpret the works of eighteenth-century and early nineteenth-century German philosophers and biologists.

According to the classical model, a proper science is a system *S* satisfying the following conditions:

- All propositions and all concepts (or terms) of *S* concern a *specific set of objects* or are about a *certain domain of being*(*s*).
- There are in *S* a number of so-called *fundamental concepts* (or terms).
- All other concepts (or terms) occurring in *S* are *composed of* (or are *definable from*) these fundamental concepts (or terms).
- There are in *S* a number of so-called *fundamental propositions*.
- All other propositions of *S follow from* or *are grounded in* (or *are provable* or *demonstrable from*) these fundamental propositions.
- All propositions of *S* are *true*.
- All propositions of *S* are *universal* and *necessary* in some sense or another.
- All propositions of *S* are *known to be true*. A non-fundamental proposition is known to be true through its *proof* in *S*.
- All concepts or terms of* S* are *adequately known*. A non-fundamental concept is adequately known through its composition (or definition). (de Jong and Betti 2010, p. 186)

We will argue that at least two major contributors to the emergence of biology in Germany, Caspar Friedrich Wolff and Gottfried Reinhold Treviranus, sought to provide a conception of the new science of life that satisfies these criteria, and that they did so because they endorsed some variety of the classical model. We will show that both Wolff and Treviranus took over strong commitments to the axiomatic model from influential philosophers of their time, Christian Wolff and Friedrich Wilhelm Joseph Schelling, respectively. An important contribution of our paper is that we show that, although Wolff and Treviranus engaged in empirical and experimental science, this does not imply that they did not accept a deductive and axiomatic model of science. Rather, both authors, following Christian Wolff and Schelling, tried to *combine* experimental science with a deductive mode of presentation by showing that experimentally established empirical propositions can be deductively ordered in an axiomatic system. We will further show that, in the works of C.F. Wolff and Treviranus, the axiomatic conception of science was coupled with the attempt to provide life sciences with fundamental concepts and fundamental propositions that were specific to the life sciences and not reducible to concepts and propositions of other sciences such as physics. This attempt was a necessary step in the emergence of biology as an autonomous proper science.

We first discuss Christian Wolff’s employment of the classical model. Next, we provide historical context that demonstrates the influence of the axiomatic ideal of science on eighteenth-century German philosophy and science, while also discussing debates on the axiomatic ideal. We then show, drawing on the work of Shirley Roe, how Christian Wolff’s conception of science motivated C.F. Wolff’s methodology in his biological works. Then we argue that the axiomatic conception of science lived on in the work of Johann Gottlieb Fichte and Friedrich Wilhelm Joseph Schelling and shaped the latter’s conception of *Naturphilosophie*. Finally, we argue that the axiomatic ideal expressed in Schelling’s *Naturphilosophie* underlies core aspects of Treviranus’s approach in his *Biologie.* Our arguments will show that, for such authors, the new science of biology envisaged in the eighteenth and early nineteenth century was not just supposed to be a naturalist explanatory account of biological phenomena, but also a hierarchical system of propositions and concepts about a specific domain of objects, where the specificity of the domain and of the science are guaranteed by the existence of certain fundamental concepts and axioms concerning “life” or “organic body.”^1^

## Christian Wolff’s Axiomatic Method

### Christian Wolff’s Axiomatic Physics

Christian Wolff (1679–1754) claimed that sciences should be treated in accordance with the mathematical method. Wolff’s mathematical method is an instantiation of the classical model (de Jong and Betti 2010). It prescribes that all non-fundamental concepts of a science must be defined on the basis of fundamental concepts and that all non-fundamental propositions of a science are grounded in fundamental propositions (see Zedler 1739, p. 2053; on Wolff’s mathematical method, see Tutor 2018). Although the mathematical method and the classical model of science should not be identified—the former being a particular *variety* of the classical model—Wolff can be said to be a follower of the classical model. That Wolff adopted an axiomatic ideal of science is also evident from the following definition of science, given in the *Preliminary Discourse*:

By science here I mean the habit of demonstrating propositions, i.e., the habit of inferring conclusions by legitimate sequence from certain and immutable principles. (C. Wolff [1728] 1963, p. 17)

Wolff defines science as the ability to *demonstrate* conclusions from first principles (capturing conditions (3a) and (3b) of the classical model) and notes that the first principles are *certain*, which implies that the conclusions deduced from them are certain too. According to Wolff, a proposition is *certain* if (i) it is true, and (ii) it is known to be true (Madonna 1987, p. 19). Hence, this definition also captures conditions (4) and (6) of the classical model. Having established that Wolff adopted an axiomatic ideal of science, we will consider how Wolff’s ideal shaped his treatment of physics.

Wolff’s physics is articulated in the *Vernünfftige Gedancken von den Würckungen der Natur* from 1723 (*Deutsche Physik*) and the *Allerhand Nützliche Versuche* from 1721–1722 (*Deutsche Experimentalphysik;* on Wolff’s physics, see de Angelis 2018). The first is a work in what Wolff calls dogmatic physics, and the second a work in experimental physics. Experimental physics employs the *analytic method*, reasoning from effects to their causes, whereas dogmatic physics employs the *synthetic method*, reasoning from causes to their effects.^2^ A function of experimental physics is to discover the fundamental principles of physics: “That part of philosophy, which experimentally establishes the principles of physics and which illustrates what is treated in physics, is called experimental physics” (C. Wolff [1728] 1963, pp. 53–54).

To understand the method of experimental physics, it is good to start with an example. In his *Deutsche Experimentalphysik*, Wolff described an experiment that shows that air has an expansive force. The experiment consists of placing a bladder filled with air under a glass jar and removing the air surrounding the bladder through an air pump (van den Berg 2014, p. 31; Anderson 2005, pp. 39–40). This results in the expansion of the bladder. Since the bladder contains nothing but air, it must be the air in the bladder that expands the bladder when the surrounding air is removed. From this Wolff concludes that air must have an expansive force (C. Wolff [1727] 1982, pp. 130–131).

In the *Deutsche Logik*, Wolff explains how the experimental finding that air has an expansive force can be proved in accordance with the mathematical method (C. Wolff [1754] 1978, pp. 176–178; for reconstructions, see van den Berg 2014, pp. 31–33; Anderson 2005, pp. 39–40). He intends to give an *analytic* syllogistic demonstration of the proposition that air has an expansive force, reasoning from the consequences to the grounds, and thus formalizes the reasoning behind the experiment. Wolff articulates the following proof, which is a reconstruction of the proof that Wolff himself provided in syllogistic form in his *Deutsche Logik* (C. Wolff [1754] 1978, pp. 176–178)^3^:

1. What continuously endeavors to expand has an expansive force [definition of expansive force].
2. What begins to expand when resistance is removed continuously endeavors to expand [proposition based on experience].
3. Hence, what begins to expand when resistance is removed has an expansive force [conclusion from (1) and (2)].
4. What expands a bladder when resistance is removed must also expand itself [proposition based on experience].
5. The air expands the bladder when resistance is removed [from the experiment].
6. Hence, the air must expand itself, when resistance is removed [conclusion from (4) and (5)].
7. What begins to expand when resistance is removed has an expansive force.
8. The air begins to expand when resistance is removed.
9. Hence, the air has an expansive force.[Q.E.D.]

Wolff’s proof is a deductive reconstruction of a proposition that was proved *experimentally* in his *Deutsche Experimentalphysik*. Hence, Wolff combines experimental research with a deductive mode of presentation.^4^ The premises of his syllogistic proof are definitions based on experience and propositions of experience. Hence, Wolff’s premises are definitions and *empirical* propositions. In what sense is Wolff’s method a priori? The deductive relations between the propositions are a priori insofar as the theorems follow deductively and a priori from the premises. Hence, the *connection* between the propositions is a priori. However, the discovery of these propositions and the content of the propositions is not a priori: we are dealing with empirically discovered propositions with empirical content.^5^

If we turn to Wolff’s *Deutsche Physik*, that is, his dogmatic physics, we see that the propositions that Wolff derived in his experimental physics function as fundamental principles (axioms) of dogmatic physics. Thus, in his account of air in the *Deutsche Physik*, Wolff specifies the axioms, proved in his experimental physics, that air has weight and that air has an expansive force (C. Wolff [1723] 2003, p. 272). On the basis of these axioms, he proves synthetically, reasoning from the causes to the effects, that the air must surround the entire earth, that air must penetrate and fill the spaces existing within bodies, and many other propositions (C. Wolff [1723] 2003, pp. 272–274). Thus, Wolff combined the analytic and synthetic method. Wolff valued experiments as much as he valued deductions from first principles. In physics, we must strive for *harmony between reason and experience*. This means that when we prove a proposition we must provide (a) a demonstrative proof of this proposition from principles, and (b) corroborate the proposition by experiment (de Angelis 2018, p. 340).

Wolff’s dogmatic physics has (i) fundamental propositions and (ii) non-fundamental propositions demonstrated on the basis of the fundamental propositions. In other words, Wolff’s dogmatic physics satisfies conditions (3a) and (3b) of the classical model of axiomatic science. Physics is also an axiomatic science insofar as its propositions are *certain*, that is, (i) true and (ii) known to be true. We have seen that it follows from Wolff’s general definition of science that the propositions of a science must be certain. In the *Preliminary Discourse*, Wolff similarly notes that philosophy in general, which includes physics as a part, must “possess complete certitude” and that certain propositions result from demonstration from certain principles (C. Wolff [1728] 1963, p. 18).

How is certainty arrived at in physics? This happens through analytic and deductive demonstrations from certain premises that express experience. Wolff states that demonstrations can be based on premises that express *clear experience* (C. Wolff [1754] 1978, p. 172). We have given an example of a demonstration based on experience with our analysis of Wolff’s proof of the proposition that air has an expansive force. This analytic demonstration proceeded from propositions based on certain experiments and experience, thus yielding certainty.

Wolff recognized that sometimes in physics we are confronted with *probable* as opposed to certain propositions, such as hypotheses (Madonna 1987, p. 30). However, this does not mean Wolff gave up his ideal of *certain* science, as Madonna argues. Such a reading is incompatible with Wolff’s definitions of science, which, as we have seen, stress that propositions must be certain. Moreover, as de Angelis notes (2018, p. 339), in his *Deutsche Experimentalphysik* Wolff notes that the goal of physical enquiry is *certainty* (C. Wolff [1727] 1982, Vorrede). We submit that Wolff viewed his axiomatic ideal of science as an ideal that was not always realized in scientific practice. His attempt to construct an axiomatic physics was an attempt to ground physics as a proper science. He recognized that hypotheses were used in science, but this meant that sciences that used hypotheses were not (yet) proper sciences. This interpretation is confirmed by the fact that Wolff remarks that hypotheses do not yield proper knowledge but mere *opinio* (C. Wolff [1740] 1983, II, p. 448).

## Christian Wolff and the Life Sciences

Wolff’s methodological reflections on the life sciences, contained in his *Deutsche Physik*, are based on his metaphysics.^6^ In his metaphysics, Wolff argued that the mode of composition of the parts of a composite body constitutes its essence. The essence of a body provides the ground for knowing why a body has certain attributes (C. Wolff [1751] 2003, pp. 18–19, 23; van den Berg 2014, p. 61). On the basis of these metaphysical views, Wolff articulates a methodology for investigation in the life sciences (C. Wolff [1723] 2003, pp. 600–601; van den Berg 2014, pp. 66–69). This methodology prescribes that one (i) has knowledge of the parts of organisms (and of the parts of parts, etc.), and (ii) has knowledge of the mode of composition of these parts (and of the parts of parts, etc.). In this way, we gain cognition of the essence of organisms and we can specify the proper *grounds* of organic processes, such as growth and nutrition. According to Wolff, anatomy is one of the fundamental sciences upon which our cognition of organisms is based (C. Wolff [1723] 2003, pp. 600–602), since anatomy provides us with knowledge of the parts of organisms and their mode of composition.

Wolff follows these methodological guidelines when discussing the growth of plants. He first identifies the parts of every plant and their mode of composition. He then identifies the parts of these parts and their mode of composition. For example, he argues that the root of a plant consists of (x) the bark, (y) the wood, and (z) the pith. The bark of the root (x), in turn, consists of (A) a membrane, and (B) a spongy substance named *parenchyma* (C. Wolff [1723] 2003, pp. 603–613).

On the basis of this anatomical analysis, Wolff explains the processes of nutrition and growth of plants. He identifies water as a source of nutrition of plants and explains how water is absorbed by the roots. Wolff notes that (A) the membrane and (B) the parenchyma of the bark of roots (x) explain the absorption of water: the membrane attracts and makes the passage of water possible and the parenchyma absorbs water (C. Wolff [1723] 2003, pp. 603–605, 626–627; van den Berg 2014, p. 67). In order to explain the absorption of water by the roots, Wolff appeals to physical laws established in his experimental physics. Wolff explains absorption as follows: the root of a plant lies under the surface of the soil. When the soil is wet, it contains droplets of water (van den Berg 2014, p. 67). The bark of a plant root contains a large amount of air. When the earth is wet the droplets of water move downward, due to the physical law that the weight of water is greater than that of air, while the air in the bark will move upward. Hence, from knowledge of (i) the parts and subparts of roots, (ii) their relationships, and (iii) physical laws, we gain knowledge of the absorption of water by roots (C. Wolff [1723] 2003, pp. 626–627; van den Berg 2014, p. 68).

Wolff did not consider the life sciences to be sciences distinct from physics. He does not delimit what we now call *biology* from other sciences by insisting it has a special domain of investigation, nor does he specify fundamental concepts and principles of biological research. The life sciences are treated as parts of physics that are based on the fundamental physical laws established by experimental physics. Hence, according to Christian Wolff, life sciences are forms of applied physics.^7^

To conclude: Christian Wolff conceived of the life sciences as parts of natural science that must be treated axiomatically. Wolff’s method is axiomatic in the sense that explanations in the life sciences must proceed from physical laws of nature (the axioms of physics). According to Wolff, explanations in the life sciences are given on the basis of anatomical propositions and on the basis of physical laws. Hence, non-fundamental propositions concerning organic phenomena are deduced from the fundamental propositions of physics. However, what we now call biology is not treated as an autonomous science.

## The Ideal of Axiomatic Science in Eighteenth-Century German Philosophy and Science

In this section, we show that the axiomatic ideal of science, as codified by the classical model, was influential in eighteenth-century German philosophy and science. We show that several figures in German philosophy and science accepted the ideal, even if they adhered to different varieties of it. We further describe debates on the axiomatic ideal. These debates concern the scope of the axiomatic ideal, that is, the extent to which different sciences could and should satisfy the ideal.

### The Ideal of Axiomatic Science in Eighteenth-Century German Philosophy

That the axiomatic ideal of science, as codified by the classical model, was widespread throughout the history of philosophy and science has been stressed by de Jong and Betti (2010). Recent research has shown that it was influential in eighteenth-century philosophy. Willem de Jong and Hein van den Berg have argued that Immanuel Kant was an adherent of the classical model (de Jong 1995, 1997, 2010; van den Berg 2011, 2014, Chap. 2). Our group is currently writing a paper in which we use a large corpus in order to quantitatively determine the spread of the axiomatic ideal of science in eighteenth-century German logic and philosophy. Early results suggest that acceptance of (parts of) the classical model was common. It is only through such large-scale and quantitative investigations, we believe, that we can specify how dominant the axiomatic ideal was. In this paper, we cannot settle such issues and restrict ourselves to discussion of selected authors.

As Betti and van den Berg (2014) stress, the classical model articulates the core of an axiomatic ideal of science. However, different authors accepted different *varieties* of the classical model. By describing how a core ideal of axiomatic science was differently instantiated by different authors, we can describe discontinuities within continuities. Thus, Wolff, Georg Friedrich Meier (1718–1777), Johann Heinrich Lambert (1728–1777), and Kant all accepted the idea that a science should have fundamental propositions or judgments that ground non-fundamental propositions or judgments.^8^ However, these authors differed on how we justify the fundamental propositions or judgments. For example, Wolff thought that some axioms or postulates of sciences are derived from definitions (C. Wolff [1750] 1999, I, p. 16, [1754] 1978, p. 161), whereas Lambert and Kant rejected this idea, and thus rejected Wolff’s mathematical method, which is a specific *variety* of the classical model.^9^ Thus, although Wolff, Meier, Lambert, and Kant all accepted the classical model, they accepted different varieties of the model.

An important and, as we will see, influential variety of the axiomatic ideal of science was the one adopted by Kant. In his *Metaphysische Anfangsgründe der Naturwissenschaft* (1786), Kant wanted to ground physics as a proper science by providing the a priori principles of physics (van den Berg 2014; Friedman 1992a). This was an attempt to ground physics as an axiomatic (proper) science and to secure that the propositions of physics were *apodictically certain* (van den Berg 2014, Chap. 2; 2011).^10^ Like Wolff, Kant acknowledged that sciences make use of probable hypotheses. However, he remained committed to the ideal of apodictic certainty. He argued that hypotheses can be *transformed* into certain propositions. For example, Kant stated that the Copernican hypothesis was transformed into a certainty because it followed from the laws of motion (Kant 1998, KrV, Bxxii). Here, Kant is referring to the fact that Newton, in book III of the *Principia*, provided definite justification for the Copernican hypothesis (Friedman 1992a, pp. 142, 170). Thus, on the Kantian variety of the axiomatic ideal of science, hypotheses can be transformed into certainties once they are embedded in a system of a priori grounded physical laws.

## Debates on the Ideal of Axiomatic Science in Eighteenth-Century German Philosophy and Physics

In this subsection we discuss eighteenth-century debates on the scope of the axiomatic ideal of science. We can start identifying conceptions of science in eighteenth-century Germany by considering philosophical and scientific dictionaries, lexica, and encyclopedia. In 1726, the German philosopher and theologian Johann Georg Walch (1693–1775) published the *Philosophisches Lexicon*, which contains a lemma on science (see Klemme and Kuehn, pp. 829–831). Walch (1726, p. 2919) notes that the term *science* can be taken in two senses: (a) as applying to any body of cognition, whether it is *certain* or *probable*, or (b) as applying to a doctrine of truths that are *certain*, which is called *scientia*. Walch associates certainty with demonstration: it is through inference from certain principles that we obtain certainty (1726, p. 2920). In short: *certainty* was what distinguished *scientia* from science in a broad sense. Since in the Wolffian tradition certain cognition was taken to be cognition that is *true* and *known to be true*, and since Walch also associates certainty with demonstration from principles, we submit that *scientia* corresponds to what we call the axiomatic ideal of science. Walch notes, however, that there is debate whether physics constitutes *scientia* (1726, p. 2920). Thus, he states that Aristotle, Descartes, and Gassendi took physics to be *scientia*, whereas modern authors, such as the philosopher and physicist Johannes Andreas Rüdiger (1673–1731), denied that physics is *scientia*. In 1748, parts of Walch’s article on science were reprinted verbatim in Johann Heinrich Zedler’s *Grosses vollständiges Universal-Lexicon aller Wissenschaften und Künste* (1748), which shows that the distinction between *scientia* and a broader conception of science remained pertinent.

In 1728 (2nd ed., 1733), the logician August Friedrich Müller (1684–1761) published his *Einleitung in die philosophischen Wissenschaften.* In this book, Müller claimed that some sciences can be treated demonstratively, whereas other sciences must be treated in accordance with the rules of probability (1733, p. 223). As he considers physics to be a science that must be treated in accordance with the rules of probability, Müller argues that in physics we cannot achieve certainty (1733, p. 638). Müller thus claimed that physics is not *scientia* and was sceptical about the scope of the axiomatic ideal.

Another philosopher who argued that physics does not satisfy the ideal of *scientia* is Christian August Crusius (1715–1775), a German philosopher who criticized Wolff (Klemme and Kuehn 2016, pp. 149–154). If we look at Crusius’s conception of science, as recorded in his *Weg zur Gewißheit und Zuverläßigkeit der Menschlichen Erkenntniß* (1747), it is clear that he accepts some aspects of the axiomatic ideal. Thus, he accepts that a science must have fundamental concepts in terms of which we define non-fundamental concepts, and must have fundamental propositions that ground non-fundamental propositions (Crusius 1747, pp. 865, 475, 68–79). However, Crusius claims that the philosophical sciences contain *probable* propositions (1747, pp. 78–79; on physics, see Crusius 1749, pp. 23–24, 54, 59), which seems difficult to reconcile with the axiomatic ideal that the propositions of a science must be *certain*, that is, that they must be true and *known* to be true.

Christian Wolff thought that in science we infer probable propositions on the basis of *deductive* arguments based on *probable premises* (Cantù 2018, p. 370 n. 64). Crusius followed this conception of probability (1747, pp. 489–490, 639–641). Hence, we can have a deductively ordered science, where non-fundamental propositions are deduced from more fundamental propositions, (some of) the more fundamental propositions of a science are *probable*, and consequently (some of) the non-fundamental propositions are probable too. If we look at Crusius’s writings on physics in his *Anleitung über natürliche Begebenheiten ordentlich und vorsichtlich nachzudenken* (1749), we see that Crusius thinks of physics as a deductively ordered science in which we prove non-fundamental propositions from more fundamental propositions, but some of the more fundamental propositions of which are *probable*. Crusius calls the probable principles of physics *presumptions* (Crusius 1749, p. 60; 1747, p. 711). He provides many examples of principles in physics that are presumptions (1749, pp. 66–67), such as, for example, the proposition that “nature takes the shortest road” (1749, pp. 79–80).

To conclude: the axiomatic ideal of science, called *scientia*, was recognized in eighteenth-century German philosophy. It was contrasted to a broader conception of science that was applied to any systematic body of cognitions. Debate on the axiomatic ideal of science mainly concerned its scope and centered on the role of probabilities in science.

### The Ideal of Axiomatic Science in Eighteenth-Century German Physics and Buffon’s Philosophy of Science

In this subsection, we show that elements of the axiomatic ideal of science were taken up in eighteenth-century German textbooks on physics. This proves the enduring influence of the axiomatic ideal, although, again, not all authors thought the axiomatic ideal could be applied to physics. We also briefly discuss the work of the biologist Buffon.

A Wolffian presentation of physics is given by the physicist Johann Peter Eberhard (1727–1779) in his *Erste Gründe der Naturlehre* (1753) (Klemme and Kuehn 2016, p. 176; on Eberhard, see van den Berg 2014, pp. 160–163; Pollok 2001). Eberhard distinguishes between two methods in physics: (i) the synthetic and a priori method, which derives consequences through correct inferences (*richtige Schlüsse*) from first principles, and (ii) the analytic and a posteriori method, which derives knowledge on the basis of experience (Eberhard 1753, p. 4). Physics combines these methods. From observation and experiments we must (analytically and a posteriori) derive laws of nature and then subsequently, synthetically and a priori, derive consequences from the laws of nature (Eberhard 1753, p. 5). This distinction of methods corresponds to the two types of method that Wolff adopted in his experimental and dogmatic physics. If we look at Eberhard’s a priori method, we see that, like Wolff, he uses certain metaphysical principles, definitions, and propositions based on experience to construct deductive demonstrations in physics. For example, Eberhard starts his physics by stating *empirical* propositions about bodies: bodies are (i) extended, (ii) impenetrable, etc. (1753, p. 7). He then gives a *definition* of space, a *definition* of place, and argues on the basis of (a) Leibniz’s law of identity and (b) the claim that bodies are impenetrable whereas space is not, that space and body are distinct (pp. 7–9). In a later edition of his textbook, Eberhard argues that he wants to establish physics as a system based on principles (van den Berg 2014, p. 162), thus ascribing to the ideal of axiomatic physics (Eberhard 1774, pp. 162–163). Eberhard also adopted Wolff’s method of seeking harmony between reason and experience. He provided deductions of propositions from principles, which he then also corroborated by experiments (1753, p. 11).

The ideal of axiomatic physics thus had an impact on eighteenth-century German physics. However, there were authors who departed from this ideal. For example, Michael Christoph Hanov (1695–1773), a student of Wolff who was one of the first to introduce the term *biology* for a branch of science (McLaughlin 2002), published the first volume of his *Philosophiae naturalis, sive, Physica dogmaticae* in 1762. This work follows the axiomatic ideal in a number of respects. Hanov, like Eberhard, argues that in physics we can reason analytically or a posteriori from the consequences to the grounds or principles, or we can deduce the consequences a priori (synthetically) proceeding from the empirically established principles (Hanov 1762, p. 13). Hence, the method that Hanov adopts is identical to that adopted by Wolff. However, Hanov adopts a broad conception of science insofar as he argues that highly probable knowledge counts as science. A similar conception of science is formulated by the natural historian and professor of medicine Johann Christian Polykarp Erxleben (1744–1777), who wrote his *Anfangsgründe der Naturlehre* in 1772. In this work, Erxleben accepted part of the axiomatic model when he construed *explanations* as correct inferences from natural laws that show that something must necessarily be the case (Erxleben 1772, p. 5). However, he also noted that in physics we must often resort to probable hypotheses (1772, pp. 5–6).^11^ A similar stress on the necessity of probable hypotheses in natural science is articulated at the end of the eighteenth century in Gehler’s *Physikalisches Wörterbuch* (Gehler 1789, II, pp. 675–679). Hence, there are authors who seem to argue that physics cannot satisfy the axiomatic ideal. However, as mentioned, there were also people, such as Kant, who wanted to transform physics into an axiomatic science. Finally, we may note that Zammito (2017, pp. 486–488) has argued that at the end of the eighteenth century we see the rise of a specific concept of *Naturwissenschaft* as an empirical, experimental, and non-mathematical science, and that several practicing scientists were skeptical of the traditional ideal of *scientia* or axiomatic science, and in particular of Kant’s restrictive notion of proper science. This is no doubt true, but, as we shall see, the axiomatic ideal of science continued to exert influence on some scientists.

We conclude by discussing the concept of science offered by the influential eighteenth-century French natural historian Georges-Louis Leclerc, Comte de Buffon (1707–1788), which probably contrasts with the axiomatic ideal. The dominant interpretation of Buffon is that he rejected the demonstrative ideal of science (Sloan 2006, p. 630; Zammito 2018, p. 111; on Buffon’s scientific method, see also Hoquet 2010). For Zammito, Buffon is someone who adopted experimental Newtonianism, a position Zammito also ascribes to other naturalists such as Albrecht von Haller (1708–1777), who was a critic of Christian Wolff (Zammito 2018, p. 69). What makes this interpretation attractive is that it fits the scientific practice of Buffon and von Haller. For example, in Buffon’s *Histoire naturelle* (1749–1804), we find many inductive and analogical arguments, and it is difficult to find any demonstration from first principles (on analogy in Buffon, see Reil 2005, pp. 55–56; van den Berg 2018, pp. 70–71). This shows that the life sciences, as they were practiced in the eighteenth century, were not axiomatic sciences. However, we argue that some philosophers and scientists felt it was a problem that the life sciences did not fit the axiomatic ideal. Because these people accepted the axiomatic ideal, they wanted to transform the life sciences into axiomatic sciences. Thus, we should not project the experimental Newtonianism of von Haller, Buffon, and others onto all life scientists in the eighteenth century.

## Caspar Friedrich Wolff’s Axiomatic Embryology

### *Deductive Proof in the* Theoria generationis *(1759)*

Shirley Roe (1979) argued that Christian Wolff’s philosophy of science influenced the methodology of Caspar Friedrich Wolff (1733–1794). We build on and further develop Roe’s interpretation. Wolff was educated at the University of Halle, 1754–1759 (Roe 1979, p. 13). Halle housed a significant number of Wolffians, although Christian Wolff’s influence waned over the course of the eighteenth century (Albrecht 2018, pp. 430–439). The Wolffian physicist Johann Peter Eberhard (1727–1779), whom we have discussed, became professor of philosophy at Halle in 1753 and of medicine there in 1756 (Klemme and Kuehn 2016, p. 176). Hence, at the time when Caspar Friedrich Wolff attended Halle’s medical faculty, there were Wolffian scientists working there, although he was of course exposed to Newtonian influences there as well (Jahn 1998–1999).

In his dissertation *Theoria generationis* (1759), C.F. Wolff developed an epigenetic theory of generation.^12^ As Roe emphasized, Wolff’s *Theoria generationis* was written in accordance with Christian Wolff’s mathematical method:

One of the most striking aspects of Wolff’s writings, particularly the *Theoria generationis*, is their scholastic, deductive style of presentation. Wolff’s dissertation is a model of the “mathematical method” championed by Christian Wolff .... The *Theoria generationis* extends this method to embryology through a deductive scheme based on principles, definitions, scholia, and syllogistic reasoning. (Roe 1979, pp. 13–14)

Roe is correct to stress the impact of Christian Wolff’s axiomatic ideal of science on C.F. Wolff. However, her interpretation has been challenged by Zammito (2018, p. 155). We will return to Zammito while defending Roe’s interpretation below; here, we remark that we do not think that Roe sufficiently stressed the implications of Wolff’s axiomatic method. According to our reading, C.F. Wolff’s axiomatic treatment of embryology has the purpose to present embryology as a proper axiomatic science that conforms to Christian Wolff’s ideal of science. We argue that the axiomatization of embryology provided a small step in the emergence of biology as a special science.

Caspar Friedrich Wolff’s adherence to an axiomatic ideal of science becomes clear in his introductory remarks to the *Theoria generationis*. There, Wolff construes scientific explanations as deductive demonstrations from principles or laws. He argues that one only explains generation if one “deduces the parts of bodies and the mode of composition of these parts from principles or laws” (C.F. Wolff [1759] 1999, p. 4; Roe 1979, p. 14). Wolff can thus be said to accept conditions (3a) and (3b) of the classical model. Wolff further notes that in his theory of organic generation we specify the true causes of organic bodies, and thus are provided with philosophical, as opposed to mere historical, cognition of these organic bodies (C.F. Wolff [1759] 1999, p. 5). Here, Wolff employs the distinction between historical and philosophical cognition introduced by Christian Wolff (C. Wolff [1728] 1963, pp. 3–6; Roe 1979, p. 14). Historical knowledge is descriptive empirical knowledge of the world. It provides bare knowledge of facts and merely shows *that* something is the case. In contrast, philosophical knowledge is knowledge of the reason of things that explains *why* something is the case. C.F. Wolff attempts to provide philosophical knowledge of organic bodies: he explains *why* organic bodies are structured as they are. Wolff also claims that in his work he *demonstrates* truths from principles. Thus, for example, he claims that he has demonstrated growth from its principles, *ex suis principiis demonstrata* (C.F. Wolff [1759] 1999, p. 88). Christian Wolff reserved the term *demonstration* for deductive inferences proceeding from certain principles (C. Wolff [1754] 1978, p. 172). C.F. Wolff’s use of the term *demonstration* suggests, given his frequent use of Wolffian terminology, that he also views explanations as deductions from principles. Finally, C.F. Wolff states that a theory is *true* if one first *demonstrates* the principles and laws of the theory and then subsequently shows that from the principles the consequences *necessarily follow* (C.F. Wolff [1759] 1999, p. 4). This claim indicates that Wolff wanted to present his theory in a deductive axiomatic fashion. It also suggests that C.F. Wolff adopted Christian Wolff’s combined demonstrative analytic and synthetic method. We argue that this is indeed the case.

Through providing a philosophical account of the structure of organic bodies, C.F. Wolff’s theory of organic generation provides us with what he calls “the science of the organic body” (C.F. Wolff [1759] 1999, p. 5). This shows that he wanted to ground embryology as a proper axiomatic science. In the next section, we will argue that the axiomatic treatment of embryology provided a small step in the emergence of biology as a special science.

### Caspar Friedrich Wolff’s Account of Nutrition in Plants

We now turn to the question of how the axiomatic ideal influenced C.F. Wolff’s scientific theorizing. To achieve this end, we will sketch Wolff’s theory of organic generation. As Roe argued, Wolff presented a theory of the generation of plants and animals that was based on two factors: (i) the ability of plant and animal fluids to solidify, and (ii) a force called the *vis essentialis* (1979, pp. 5–6). The origin and formation of plants and animals are brought about by the secretion of fluids that solidify into structures. In animals, for example, the first structure that solidifies is the spinal column, which subsequently secretes, for example, the wings and legs (1979, p. 6). This theory of organic generation was based in part on Wolff’s account of nutrition and growth in plants and animals. Wolff took nutrition, growth and reproduction to be analogous processes (C.F. Wolff [1759] 1999, p. 6). Hence, an account of, for example, nutrition and growth, will shed light on the analogous process of reproduction. In the following, we will discuss Wolff’s account of nutrition in plants. This shows how Wolff attempted to treat embryology demonstratively.

Wolff’s account of nutrition in plants follows the analytic method, that is, he reasons demonstratively from consequences to their causes (just like Christian Wolff’s deductive demonstration of the proposition that air has an expansive force was an analytic inference). That a large part of Wolff’s work adopted the analytic method is explicitly stated in paragraph 71 of the *Theoria generationis*, where he remarks that he wanted to discover the *principles* and *laws* of generation a posteriori (C.F. Wolff [1759] 1999, p. 44; Roe 1979, p. 16). At the start of providing his account of nutrition in plants, Wolff introduces his famous *vis essentialis*. Wolff argues that plants take up nutritious fluids, that these fluids are distributed throughout the plant, and that these fluids evaporate. According to Wolff, there must be a force that is responsible for taking up fluids from the surrounding environment and that distributes these fluids throughout the parts of the plants. This force is the *vis essentialis* (C.F. Wolff [1759] 1999, pp. 11, 12). The *vis essentialis* is later called a principle that allows one to demonstrate or explain phenomena (C.F. Wolff [1759] 1999, p. 44).

Having described the *vis essentialis*, Wolff turns to a discussion of the channels through which fluids move in plants. Wolff notes that, when one places a root or a branch under a microscope, one observes vessels (*Gefäße*, *vasa*) and cylindrical fluid droplets contained in these vessels. Hence, these vessels are the primary means through which fluids move. However, in young leaves one does not find vessels: they merely contain vesicles (*Bläßchen*, *vesiculae*). Since these parts of the plants must also receive nutrients, Wolff concludes that the fluids move through the substance of vesicles and disperse throughout the plant. In other words, fluids move through the solid parts of plants with the same ease as they move through vessels. Wolff concludes by providing some remarks on the growth of leaves and roots of plants. He claims that leaves grow by new vesicles inserting themselves between old vesicles or by vesicles expanding, whereas roots grow by the insertion of new vessels between old vessels or by vessels expanding. Fluids must penetrate these vesicles and vessels as well as the spaces between the vesicles and the vessels (C.F. Wolff [1759] 1999, pp. 12–14).

After discussing the trajectory of nutritious fluids in plants, Wolff provides a comparison between the vessels and vesicles in young (parts of) plants and older (parts of) plants. He notes that in young plants, the vesicles are moveable parts that can be designated as pores or cells, whereas the vessels are moveable parts that can be designated as canals. Moreover, in young plants the substance that exists between vesicles and vessels is a homogeneous soft substance that is permeable. In older plants, this substance has solidified. According to Wolff, this solidification process is responsible for the growth of plants, which, as we have seen, occurs by new vesicles inserting themselves between old vesicles and through the insertion of new vessels between old vessels. Wolff argues that the soft substance that exists in the spaces between the vesicles contains a fluid that expands and solidifies and forms a new vesicle. Likewise, the soft substance that exists between the spaces of vessels contains a fluid that expands and solidifies to form new vessels. Solidification of nutritious fluids is thus the process through which the growth of plants is explained (C.F. Wolff [1759] 1999, pp. 16–17). Here we see that the ability of plant and animal fluids to solidify, which was used by Wolff to explain the generation of plants and animals, was already introduced by Wolff in his account of nutrition and growth in plants.

In summary, Wolff has argued that (i) the *vis essentialis* is responsible for distributing nutritious fluids through plants, (ii) these fluids run through vesicles and vessels as well as through the matter that exists in between the spaces of these vesicles and vessels, and (iii) the fact that nutritious fluids run through the matter that exists between vesicles and vessels causes an expansion of this matter, which, together with the solidification of the nutritious fluid, gives rise to new vesicles and vessels. Hence, nutritious fluids form new vesicles and vessels. Wolff concludes by noting that the formation of the first vesicles and vessels must also be due to the solidification of nutritious fluids (C.F. Wolff [1759] 1999, pp. 16, 20–21).

Wolff’s account of nutrition in plants is *experimental*: his conclusions follow from microscopic observation of plants. It is also meant to be a demonstrative theory. After establishing propositions experimentally, Wolff presents his argument as a deductive inference. For example, in paragraph 21, Wolff argues that: from the fact that the spaces between the vesicles and vessels are filled with soft substance (established in paragraph 20); from the fact that leaves grow through new vesicles inserting themselves between old vesicles, whereas roots grow through the insertion of new vessels between old vessels (established in paragraph 9); and from the fact that vesicles are nothing but pores or cells filled with fluids, and vessels are canals filled with fluids (established in paragraph 20), it “follows with necessity” that the soft substance occupying the space between vesicles is expanded through the fluids it contains and that vessels are formed in a like fashion (C.F. Wolff [1759] 1999, p. 17; see also Zammito 2018, pp. 157–158). The claim that propositions follow *with necessity* from each other supports the idea that Wolff is presenting a deductive inference here. Note that Wolff’s method is analytic: he reasons from the consequences to their causes. In later chapters, Wolff reverses the direction of inquiry and adopts the synthetic method, reasoning deductively from grounds to their consequences. For example, in a later chapter on the growth of plants, Wolff establishes *laws* (axioms) that govern growth and subsequently deduces particular phenomena on the basis of these laws (C.F. Wolff [1759] 1999, pp. 34, 37–38). All of this suggests that Wolff wanted to present his theory in an axiomatic fashion.

The idea that C.F. Wolff strictly followed Christian Wolff’s ideal of axiomatic science has been challenged by Zammito. Although Zammito acknowledges some influence of Christian Wolff on C.F. Wolff, Zammito writes that C.F. Wolff was an eclectic and that it is doubtful that he was “a rationalist in a sense that rendered building inferences drawn from observation and experience superfluous, or that construed rational deduction a priori as the ultimate and only `science’” (Zammito 2018, p. 154). Zammito stresses Wolff’s experimental work and contrasts this to Christian Wolff’s axiomatic or demonstrative ideal of science. Zammito offers the following justification for his reading, which is based, like our reading, on Wolff’s account of nutrition in plants:

there is no derivation a priori in this account. It is entirely a matter of experimental observation and concrete description. The first twenty propositions of part 1, then, are *entirely* empirical, not derived from axioms in some logico-deductive manner. In paragraph 21, Wolff did draw an inference that he claimed was necessary (“thus it follows with necessity”) .... I can take this only as an *inductive* inference, not a deduction, and the “necessity” Wolff claimed can only be a matter of what we would call inference to the best explanation, not demonstrative logic. (Zammito 2018, pp. 157–158)

We think Zammito’s argument is mistaken for two reasons. On the one hand, we note that Zammito is forced to explain away Wolff’s claim that his conclusion “follows with necessity.” For someone with Wolff’s philosophical background, inductive and abductive inferences do not yield “necessity”; for most authors in this period, only deductive, i.e., demonstrative, inferences necessitate their conclusion. On the other hand, we believe Zammito is led to this interpretation because he is mistaken about what the axiomatic method meant to an author like C.F. Wolff. As we argued in the previous section, Christian Wolff allowed for the possibility that some propositions in natural science are discovered experimentally and have empirical content, while insisting that after we have found these empirical propositions experimentally we must order them demonstratively. Hence, it is not the case that inferences drawn from observation and experience are superfluous within Christian Wolff’s axiomatic conception of science. C.F. Wolff adopts the same method: he first establishes propositions experimentally and then orders them deductively. We can also explain why Zammito is unable to find a synthetic argument from *axioms* in the first twenty propositions of the *Theoria generationis*. If C.F. Wolff is following Christian Wolff’s method, these first paragraphs can be read as an *analytic* part of the argument, in which Wolff reasons from consequences to their grounds and to the axioms. It is only in later chapters that Wolff adopts the synthetic method, reasoning from axioms to their consequences. Thus, as we have said, in later chapters Wolff establishes laws (axioms) that govern growth and subsequently deduces particular phenomena on the basis of these laws. We conclude, therefore, that Zammito wrongly challenges Roe’s interpretation of Wolff’s work because (1) he mistakenly believes that empirical or experimental propositions cannot play a major role in an axiomatic conception of science, and (2) he does not acknowledge that Wolff first establishes propositions experimentally and then orders them deductively. We showed that these assumptions are mistaken as assumptions about the axiomatic method proposed by Christian Wolff. As a result, the peculiarities of C.F. Wolff’s presentation to which Zammito draws our attention are entirely to be expected if C.F. Wolff is in fact following Christian Wolff’s prescriptions. We add one disclaimer: it may be the case, as Zammito’s analysis suggests, that C.F. Wolff sometimes presents what are really inductive inferences as deductive inferences. In addition, if we let a logician analyze Wolff’s work, we would no doubt find many implicit premises and leaps of inferences. However, there is ample evidence that C.F. Wolff wanted to treat embryology axiomatically, that is, that he followed Christian Wolff in accepting the *ideal* of axiomatic science. Zammito may be right that the axiomatic ideal of science is an illusion and provides a faulty conception of how science actually works (see Zammito 2017), something we know from our modern vantage point. However, this ideal did influence philosophers and scientists.

Although C.F. Wolff follows Christian Wolff’s prescriptions for science, there are important differences between the two men’s use of the axiomatic method in the life sciences. C.F. Wolff’s account of nutrition in plants is based on fundamental concepts, such as the *vis essentialis*, the notions of a vessel and vesicle, and of nutritious fluids that can solidify. On the basis of these fundamental concepts, Wolff attempts to explain nutrition in plants. As we have seen, Christian Wolff referred to general physical laws to explain phenomena such as nutrition and growth. C.F. Wolff, in contrast, refers to the action of the *vis essentialis*, which is operative in organic nature, rather than general physical laws. Hence, C.F. Wolff articulated principles that were *specific* to theorizing in the life sciences and attempted to explain organic processes in terms of these principles.

The idea that life sciences must have principles specific to these sciences was endorsed by many life scientists after Wolff. Johann Friedrich Blumenbach (1752–1840), for example, based his theorizing on the notion of a vital force. Vital forces, which were operative only in the organic domain and accounted for the vital properties of organisms, were distinguished from physical and chemical forces, to which all bodies are subject (Blumenbach 1817, pp. 16–17; van den Berg 2014, pp. 195–196). Since, according to Blumenbach, vital properties cannot be accounted for in terms of physical or chemical forces, life scientists should appeal to vital forces in order to explain the vital properties of organisms. Hence, Blumenbach introduced principles that were *specific* to the life sciences. The vital materialism that was adopted by many biologists at the end of the eighteenth century is an important factor in the emergence of biology as a special science (see Gambarotto 2018).

Finally, we may note that, in the third part of the *Theoria generationis*, Wolff specifies universal properties of organisms and universal principles of organic generation. This part contains the axioms of the life sciences. For example, Wolff specifies that a universal property of organisms is that individual parts of organic bodies cannot exist without the existence of other organic parts, or that they obtain nutrition from other organic parts (C.F. Wolff [1759] 1999, p. 145). From this fundamental principle, Wolff deduces consequences, such as the proposition that every organic body possesses a part through which the nutrition of all other parts is mediated. As an example of a fundamental principle of organic generation, Wolff mentions the familiar principle that the parts of organic bodies are formed through fluids (C.F. Wolff [1759] 1999, pp. 146, 150) which again functions as a principle from which consequences are derived. The final part of the *Theoria generationis* thus again provides evidence for our reading that Wolff first attempted to analytically discover the propositions of his theory of generation proceeding from empirical propositions, while subsequently ordering these propositions synthetically.

### Axiomatics in the Theorie von der Generation (1764) and the Emergence of Biology

In 1764, C.F. Wolff wrote a follow-up to his dissertation, called *Theorie von der Generation*, in which he again explained his theory and debates with Albrecht von Haller (1708–1777) and Charles Bonnet (1720–1793) (Roe 1979, p. 8; on the Haller-Wolff debate, see Roe 1981 and Detlefsen 2006). We will explain the impact of axiomatics on the *Theorie von der Generation* and explain the importance of Wolff’s axiomatic embryology for the emergence of biology as a special science.

As Roe has emphasized (1979, pp. 15–16), Wolff’s methodological views in his *Theorie von der Generation* (1764) are the same as those articulated in his dissertation of 1759, and show the impact of Christian Wolff’s philosophy. In the section *Begriff einer Theorie von der Generation*, C.F. Wolff again introduces Christian Wolff’s distinction between historical and philosophical cognition (C.F. Wolff 1764, pp. 8–9). Wolff’s embryology is supposed to provide philosophical as opposed to historical cognition of organic phenomena, that is, explanations for *why* organic phenomena occur. Wolff also notes that his theory of generation provides the *sufficient ground* of the parts and the composition of organic bodies (C.F. Wolff 1764, p. 11). Roe notes that the notion of a sufficient ground was used by Christian Wolff but does not explain this notion. Christian Wolff construed a sufficient ground as the set of *all* the (certain) grounds that make a proposition true (C. Wolff [1740] 1983, 2: 434–436; Madonna 1987, p. 19). Thus, the notion of a sufficient ground is tied to the idea of axiomatic science: if we have a complete demonstration of a proposition terminating in certain axioms, we know its sufficient ground. That C.F. Wolff wanted to specify the sufficient ground of organic phenomena suggests that he wanted a demonstration of these phenomena from certain principles. C.F. Wolff also again states that his theory is based on principles (*Grundsätzen*). In his debate with Haller, Wolff shows that his theory is based on true principles that explain phenomena, and he stresses the importance in his dissertation of the axiomatic presentation of his theory (C.F. Wolff 1764, pp. 32, 75, 76, 79). Finally, whereas C.F. Wolff, like Christian Wolff, recognized the role of hypotheses in science, he notes that he provides a stronger proof than proof by hypotheses (C.F. Wolff 1764, pp. 41, 63) and refers to his theory as a certain truth proved on the basis of true principles (C.F. Wolff 1764, pp. 35, 42, 60–62, 76, 111–112).

A large part of the *Theorie von der Generation* is taken up by providing experiments that provide proofs for Wolff’s theory. However, this does not indicate a departure from the ideal of axiomatic science. As we have argued, C.F. Wolff wanted to combine experimental research with an axiomatic and demonstrative presentation. Moreover, even in the *Theorie von der Generation* we can find deductive explanations that fit Christian Wolff’s ideal of science. Roe cites the explanation of the fact that animals have hearts whereas plants do not, which she presents as a deduction from experience (Roe 1979, p. 16).

There are also deductive arguments which Roe does not discuss, but which demonstrate C.F. Wolff’s debt to Christian Wolff. In Chapter 6 of the *Theorie von der Generation*, Wolff discusses what he calls imperfect leaves of plants, that is, leaves that are less developed (C.F. Wolff 1764, p. 231). When discussing the anatomy of plants, Wolff notes that there exist grades of imperfection of leaves: the leaves of the flower (*Blume*) are more imperfect than the leaves of the sepal (*Kelch*), the leaves of the pistil are more imperfect than the leaves of the flower, and so forth. Wolff calls this phenomenon the weakening of vegetation in plants. He then proceeds to provide what he calls an a priori* proof* of this phenomenon (C.F. Wolff 1764, pp. 232, 233). We have seen this terminology being used by the Wolffian physicist Eberhard, who took a priori proofs to be demonstrations from principles. This is exactly what C.F. Wolff provides, namely a demonstration based on certain and empirically established principles. He reasons as follows (C.F. Wolff 1764, pp. 234–235): (i) If (a) a plant is healthy and (b) there is a sufficient supply of nutritious fluids, a plant begins to grow and produce new parts. (ii) If there is no growth and production of new parts, or a lack of growth and production of parts, it must be the case that either (a) or (b) are not satisfied. (iii) If a plant does not grow, or there is a lack of growth and production of new parts, but is nevertheless healthy, this must be due to a lack of nutritious fluids. (iv) Consider a healthy plant, called A, that is affected by a lack of growth and production of new parts. (v) The lack of growth of A must be due to a lack of nutritious fluids, a conclusion that can, on the basis of premise (iii), be generalized to all healthy plants affected by a lack of growth and production of new plants (Q.E.D.). This is clearly meant as a deductive demonstration from principles, and it can be reconstructed as a valid deductive argument. Wolff notes that this proof secures the truth of the conclusion. However, after providing this proof, Wolff also provides experiments that support his conclusion (C.F. Wolff 1764, pp. 235, 236–243). Hence, C.F. Wolff adopted the method of Christian Wolff and Eberhard: he demonstrated truths from certain principles while also providing confirmation of these truths through experiments, striving for a *harmony between reason and experience*.

Having discussed C.F. Wolff’s main works, we may consider the excellent work of Detlefsen (2006), who, like Zammito, is sometimes skeptical of Roe’s interpretation. Detlefsen agrees that C.F. Wolff accepted Christian Wolff’s ideal of demonstration as demonstration from principles (Detlefsen 2006, p. 254). However, she also doubts Roe’s interpretation on the grounds that C.F. Wolff was an avid experimentalist. Building on the work of Duchesneau (1982, p. 330)^13^ and Rudolph (1991, p. 78), she describes C.F. Wolff’s method as a form of *experimental demonstration* (Detlefsen 2006, p. 256), because the conclusions follow from experiments. We think the term experimental demonstration is apt to characterize Wolff, because Wolff actually was an experimentalist, and we generally agree with Detlefsen’s account of experimental method in Wolff. However, we argue that C.F. Wolff also wanted to present his theory demonstratively. Remember Christian Wolff’s method: (i) in experimental physics, we construct experiments. (ii) Then, we reconstruct experimental inferences as analytic deductive arguments. (iii) Finally, we adopt the synthetic method and show how consequences follow from the axioms. C.F. Wolff clearly accepts this method. In other words, the strict dichotomy between experimentalism and a demonstrative ideal of science, which Zammito and Detlefsen seem to accept, is not apt to interpret Christian Wolff and C.F. Wolff, both of whom wanted to unify experimental science with demonstrative science.

To conclude, we discuss the importance of C.F. Wolff’s work for the emergence of biology as a science. Wolff presented embryology axiomatically, in line with Christian Wolff’s prescriptions for science. Why is this important for the emergence of biology as a science? As an anonymous referee pointed out to us, a historical-epistemological condition for the emergence of biology as a special science seems to be the idea that there is a difference between organic and physical phenomena, along with the idea that this difference applies to living nature as a whole. C.F. Wolff accepts the idea that organic phenomena must be explained by the so-called *vis essentialis*, which is operative in organic nature, and marks a difference between the physical and the organic. This is clear from his 1789 *Von der eigenthümlichen und wesentlichen Kraft*, where he argues that the essential force is peculiar to living matter (Roe 1979, pp. 21, 25, 26). However, as our referee pointed out, a stress on the difference between physical and organic phenomena was already present in Stahl’s work and was widespread in the eighteenth century (see, for example, Zammito 2018, pp. 13–36). What, then, is Wolff’s contribution to the emergence of biology? We think that it was precisely Wolff’s formal and axiomatic treatment of embryology that constituted a small step in the emergence of biology. To see this, we must again emphasize the differences between Christian Wolff’s account of the life sciences and C.F. Wolff’s views on life science.

Recall that Christian Wolff took anatomy to be the fundamental life science. For Wolff, explanations in the life sciences are based on anatomical propositions and on laws of physics. There are no fundamental concepts and propositions specific to the life sciences. For Christian Wolff, then, life sciences are not special sciences and the life sciences should be treated as forms of applied physics. C.F. Wolff differed from Christian Wolff in the following respects:

(i) C.F. Wolff specified fundamental concepts and propositions, such as the *vis essentialis*, that were specific to the organic domain. Hence, he specified true axioms of embryology, and he believed that embryology has axioms that do not reduce to physical laws (as Christian Wolff thought). This made it possible to argue that there are genuine *laws* concerning “life” that are specific to the life sciences and that life sciences are not mere forms of applied physics.
(ii) Christian Wolff’s conception of science entailed an axiomatic *hierarchy* of sciences: lower sciences are subordinated to higher sciences. C.F. Wolff’s treatment of embryology was motivated by the intention to explicate the axiomatic hierarchy of the life sciences. For Christian Wolff, anatomy was one of the fundamental life sciences. However, C.F. Wolff’s theory of generation explained why organic bodies are structured as they are and thus provided the reasons *why* certain anatomical structures occur. Accordingly, as C.F. Wolff stresses, anatomy is not the fundamental life science, embryology is (C.F. Wolff [1759] 1999, p. 5). Thus, Wolff shows that embryology provides the *foundation* of many life sciences, such as anatomy and physiology. By providing a novel account of the foundations and hierarchy of the life sciences, C.F. Wolff showed how multiple life sciences are related and can potentially be treated in a unified, systematic, and scientific way. Through his formal treatment of the life sciences, and his attempt to specify an axiom that grounds multiple life sciences, Wolff thus made a step toward the emergence of biology. As we shall see, Treviranus undertook a similar project.
(iii) C.F. Wolff took all the propositions of embryology and the life sciences to concern a specific domain of beings, namely *organic nature*. By contrast, Christian Wolff interpreted the fundamental propositions of the life sciences as physical laws concerning *matter in general* (although he may have believed that some of the non-fundamental life-sciences employ propositions that do not stem from physics). For C.F. Wolff, the formal treatment of the life sciences matched the ontological conviction that there was a difference between the organic and the physical. According to the axiomatic ideal of science, sciences are distinguished from one another through their different *domains* (see condition (1) of the classical model). C.F. Wolff’s axiomatic treatment of the life sciences allows one to distinguish the life sciences from other sciences by noting that the propositions of the life sciences concern a specific domain of organic objects, one that is distinct from the domain of objects to which the propositions of physics refer. If one accepts the axiomatic model, this is a precondition for treating biology as a special science.

To conclude: Wolff’s axiomatic treatment of embryology constituted a small step in the emergence of biology as a science. Of course, not all German biologists were concerned with treating the life sciences axiomatically. If we survey the works of Blumenbach and Johann Christian Reil (1759–1813), we see that the life sciences were not always treated axiomatically in the latter half of the eighteenth century (on Blumenbach and Reil, see Richards 2000, 2002, Chaps. 5 and 7; on Blumenbach see van den Berg 2018). However, the ideal of an axiomatic science of life did not disappear.

## The Axiomatic Conception of Science in Fichte and Schelling

By the late eighteenth century, the heydays of the Wolffian mathematical method were over (Tonelli 1959). Moreover, Wolff had lost his preeminence among German philosophers to Kant. But this does not mean that the axiomatic ideal itself had lost ground. First of all, as we indicated in “The Ideal of Axiomatic Science in Eighteenth-Century German Philosophy and Science,” Kant’s own ideal of science—as expressed, for example, in the *Metaphysische Anfangsgründe der Naturwissenschaft* (1786)—is a variety of the axiomatic ideal (van den Berg 2014, Chap. 2; Zammito 2017). Note that Kant did not follow Christian Wolff in every respect. In contrast to Wolff, he argued that some *principles* of metaphysics and natural science are synthetic a priori judgments that are justified *transcendentally*. However, once the synthetic principles and other synthetic propositions of a science are in place, we can construct sciences as axiomatic and deductive sciences (van den Berg 2014). As we shall see, Fichte and Schelling followed Kant in this line of thought. Second, an important development in post-Kantian philosophy was that several authors felt that Kant himself had not given his philosophy a scientific form. Authors such as Karl Leonhard Reinhold (1757–1823), Fichte, and Schelling felt that transcendental philosophy should be made scientific by recasting it in the form of a system. As Tom Rockmore notes, the disagreement between these authors:

did not concern the quasi-rationalist system, which was accepted as a model for system by the post-Kantians, as well as by those who influenced this aspect of the critical philosophy, such as Wolff and Lambert. All of these thinkers were clear that the concept of system entailed the rigorous interrelation of a series of propositions following from each other in a way comparable to [the] rationalist geometrical standard of deductive interrelation. (Rockmore 1989, p. 108)

In this section, we will flesh out Rockmore’s suggestion and show that Fichte and Schelling embraced the idea that a proper science should constitute a system, which means, we argue, that they were committed to the axiomatic ideal of science which they inherited from Kant. In the first subsection, we discuss Fichte’s work on the concept of science and show that he adopts a variety of the classical model that derives from Kant. In the second subsection, we discuss Schelling’s conception of science as it appears in his *Naturphilosophie* and show that it incorporates many aspects of the Kantian and Fichtean conceptions of science in a way that is clearly motivated by the axiomatic ideal of science.

### Fichte and Schelling on the Concept and the Form of a Science

Fichte’s early conception of science is presented most explicitly in his 1794 essay *Über den Begriff der Wissenschaftslehre*. There, Fichte analyzes what he takes to be an accepted concept of science and claims that it involves several conditions. The first is the following: “A Science possesses systematic form. All the propositions of a science are joined together in a first principle, in which they unite to form a whole” (Fichte 1988, p. 101; GA I, p. 38).^14^ This shows that, according to Fichte, a science must contain fundamental principles (condition (3a) of the model), that a single science should contain only one such principle, and that all other propositions should be connected to that first principle. The fact that the system needs to be grounded in a single principle is a specific feature of the variant of the classical model embraced by Fichte. That first principle, Fichte insists (1988, p. 103; GA I, p. 41), must itself be certain and cannot derive its certainty from other propositions. Fichte’s picture of the connection between this first principle and the non-fundamental propositions is as follows:

at least one proposition has got to be certain, and this proposition then, so to speak, communicates its certainty to the other propositions: so that if and insofar as the first proposition is certain, then a second proposition is too; and if and insofar as this second one is certain, then a third one is, etc. (Fichte 1988, p. 103; I, pp. 40–41)

From this picture, it becomes clear that Fichte insists on conditions (3b), (4) and (6) of the classical model, because he insists that the first principle needs to be certain, which means that it needs to be true and known to be true (conditions (4) and (6)), and that all propositions in the science are equally true and known to be true in virtue of being derivable from the first principle (conditions (4), (6), and (3b); Fichte uses terms such as *erweisen*, *beweisen*, *gründen*, and *folgern*). Tom Rockmore clarifies that “Fichte’s analysis indicates that certainty is a commutative property imparted by the deductive relation between propositions contained in a given science, which in turn rests on a primitive proposition known to be certain” (Rockmore 1981, p. 489). The relation of derivability which guarantees that certainty and truth are preserved seems to be that of logical deduction, since Fichte argues that derivability from a certain premise implies that the certainty of the derived proposition *necessarily follows* from the premise. All of this suggests that Fichte accepts the axiomatic ideal of science.

Breazeale (2001, p. 21) notes that “[i]n describing philosophy as a systematic science in which ‘all its propositions are joined together in a single first principle in which they unite to form a whole,’ Fichte seems to imply that the proper method of science is simply that of logical inference.” In a letter from January 15th, 1794 to which Breazeale refers, Fichte writes that, in philosophy, “the form of deduction is the same as in mathematics, that is, the form prescribed by general logic” (Breazeale 2001, p. 22). But Breazeale goes on to note that Fichte’s method cannot *solely* consist of logical inference, for then it would violate the Kantian stricture that “one cannot infer synthetic a priori principles directly from pure concepts” (2001, p. 22).

We agree with Breazeale that logical inference is not all there is to Fichte’s method. However, logical deduction can still play an important role in the presentation of a science according to Kant and followers of his like Fichte. The important point to understand is that once we have discovered the synthetic principles and several other synthetic propositions of a science, often by non-logical means, we can present and justify these synthetic propositions deductively. To illustrate this, consider the following Newtonian deduction, which is a simplified version of a deduction taken from Newton’s *Principia*: (i) if the motion of a body satisfies the laws of areas, the body is subject to a centripetal force. (ii) The motion of the satellites of Jupiter satisfies the laws of areas. (iii) Hence, the satellites of Jupiter are subject to centripetal forces (Newton [1726] 1999, pp. 446, 797, 802; van den Berg 2014, pp. 39–40; Cohen 1999, pp. 196, 204). This is a deductively valid argument, the premises and the conclusion of which are synthetic. The first proposition is proved mathematically by Newton and is thus, from a Kantian perspective, synthetic a priori. The second premise is what Newton calls a phenomenon based on observation and is synthetic a posteriori. From these premises the conclusion follows deductively; but that conclusion is also a synthetic and not an analytic proposition, since we cannot demonstrate it by mere conceptual analysis. This example thus shows that one can deductively order synthetic propositions after having discovered them by non-logical means (in this example, via mathematical proof and observation). Hence, Kant and Fichte can maintain that once we have the synthetic principles as well as several synthetic propositions of a science in place, we can order them in the form of a deductive axiomatic system. In this way, we can make sense of Fichte’s remarks that logical deduction has a place in the presentation of a scientific system.

The above shows that Breazeale is right to stress that Fichte’s method cannot consist of logical inference alone. After all, on Fichte’s conception, a science must contain a synthetic fundamental principle that is itself certain, and that proposition cannot be proved by deducing it from a higher principle. Discovering and justifying that principle therefore requires another, *transcendental* method. Moreover, as Breazeale rightly notes, Fichte insists on the need for reflection, imagination, and experiment in the discovery of propositions of the system (Breazeale 2001, pp. 23–26). But this, as Breazeale admits, has to do with philosophy as an *ars inveniendi*, a method of discovery. It does not commit Fichte to denying that, once we have discovered or established propositions with a method that involves, for example, experimentation, we should order those propositions in such a manner that they are all directly or indirectly deduced from the fundamental principle. Hence, we can consistently interpret Fichte as adhering to the axiomatic ideal of science while also acknowledging the importance and even indispensability of other (transcendental) methods in the construction of a science.

On the basis of the evidence presented, we can conclude that Fichte’s conception of science agrees with the classical model. Nevertheless, it is a different variety than the one found in Wolff. In the first place, it adds the requirement that a science should be based on a single fundamental principle. And in the second place, it requires transcendental proofs for at least some of its propositions.

Fichte’s conception of a science influenced Schelling’s conception of science as well. This is apparent in his early *Ueber die Möglichkeit einer Form der Philosophie überhaupt* from 1794 (Schelling 1856–1861; 1.1, p. 90), where Schelling writes that a science takes the form of a unified system, in which all non-fundamental propositions are conditioned by a single fundamental proposition. Schelling insists that the first principle of a science needs to be unconditioned within that science and that all other propositions need to be conditioned by it (constituting a series of *grounds* and *consequences*). In a footnote, Schelling suggests that, for his principle to be absolutely unconditioned, is the same as what is traditionally meant by a principle being evident or certain. Moreover, he interprets Descartes, Leibniz, Kant, Reinhold and Fichte as aiming at a deductive system (one that operates merely logically), and places himself in this tradition (Schelling 1856–1861; 1.1, p. 101).

### Naturphilosophie and the Axiomatic Method

Schelling’s conception of science and of *Naturphilosophie* builds on Kant’s account of physics as a proper science. As Beiser notes, “Like Kant, Schelling thinks that the ideal of science is a system, a complete body of propositions organized around and derived from a single principle. … It is clear that Schelling’s paradigm of knowledge and of science have their origins in Kant’s philosophy of nature” (Beiser 2002, p. 526). That Kant’s Metaphysical Foundations of Natural Science delivered the groundwork for Schelling’s *Naturphilosophie* is widely acknowledged.^15^ Schelling’s Kantian influence shows, for example, in his insistence that we need a priori principles for natural science. Furthermore, Schelling’s foundation for a *Naturphilosophie* is Kantian in inspiration because it adopts Kant’s dynamic matter theory, even though that matter theory is developed further in an original way. Finally, that Schelling, following Kant, embraced a conception of science that comes close to the axiomatic ideal of science in developing his *Naturphilosophie* was evident to early readers, such as Henrik Steffens (1773–1845), who reviewed Schelling’s *Entwurf eines Systems der Naturphilosophie*. As Zammito notes, Steffens interpreted Schelling as stating that mere empirical enquiry could only generate hypotheses. However, “the goal of knowledge was necessity … and such necessity was a philosophical, not an empirical, achievement” (Zammito 2018, p. 328). Zammito correctly states that such a view reflects the classic tradition of *scientia* in natural philosophy that was also articulated by Kant. In this section, we will argue, building on Zammito’s suggestion, that Schelling, following Kant, developed a conception of science that is a variant of the axiomatic ideal of science.

In the 1799 *Einleitung zu dem Entwurf eines Systems der Naturphilosophie*, Schelling announced that “[s]ince our investigation aims not so much toward the natural phenomena themselves as toward their ultimate grounds, and our business is not so much to deduce the latter from the former, as to deduce (*abzuleiten*) the former from the latter, our task is none other than the following: to erect a *Natural Science* in the strictest sense of the word” (Schelling 1856–1861, 1.3, p. 275; all translations of this work are ours). Here, Schelling frames the problem of *Naturphilosophie* as the search for a proper science of nature. The main criterion to which Schelling points here is that, in such a science, the phenomena should be deduced from their ultimate grounds. He insists, therefore, that proper science proceeds from first principles to phenomena and deduces the latter from the former. This reveals that Schelling has in mind conditions (3a) and (3b) of the classical model. Moreover, he insists that this deduction ought to be a priori since “knowledge in the *strictest* sense of the word is … *pure* a priori knowledge” (Schelling 1856–1861, 1.3, p. 276). The aim of Schelling’s speculative physics is thus to transform physics into a system in which all phenomena and even physical laws are ultimately deduced from a single fundamental principle so that we obtain “a science of nature a priori” (Schelling 1856–1861, vol. 1.3, p. 278).

Schelling’s insistence on a priori deduction, however, does not mean that he takes empirical information and empirical inquiry to be irrelevant, for “if in the interconnected whole of nature there is a single phenomenon that is not necessary because of this principle, or even contradicts the latter, then the presupposition itself is shown to be false, and ceases from this moment on to count as a principle” (Schelling 1856–1861, 1.3, p. 277). In his 1801 *Über den wahren Begriff der Naturphilosophie und die richtige Art ihre Probleme aufzulösen*, Schelling makes a similar point in order to insist that reason cannot contradict experience (Schelling 1856–1861, 1.4, p. 96).

Schelling warns against the misinterpretation that a science in which all sentences are a priori derived is capable of finding out all of its sentences without any mediation from experience. In fact, he says that we originally know everything through experience and that all our knowing consists of empirical propositions (Schelling 1856–1861, 1.3, p. 278). Passages such as these have been cited by scholars to stress that Schelling valued empirical inquiry (Heuser-Keßler 1986, p. 37, n. 23; Richards 2002, p. 142). But, as Robert Richards (2002, p. 142) has also noted, “Schelling believed, nonetheless, that one could cast empirically acquired knowledge into a deductive system.” In fact, Schelling describes the task of the proper science of nature as that of transforming empirical propositions into necessary propositions. Schelling clearly makes the point, which we have stressed in the previous subsection, that the fact that a (synthetic) proposition stems from experience does not mean that it cannot be derived a priori (empirical propositions can be deductively ordered). The task of a proper science is to turn such propositions into necessary ones by showing how they can be derived within an axiomatic system of propositions. Such a system, Schelling adds, “does not tolerate the hypothetical, nor the merely probable, but aims for the evident and the certain” (Schelling 1856–1861, 1.3, p. 279). Schelling therefore insists that all the propositions in a science need to be true (condition (4) of the classical model), known to be true (condition (6)), and necessary (condition (5)). In a short text from 1800, he also stresses that such evidence and certainty cannot be reached by induction and experience alone, remarking that true theories “are constructed *absolutely* a priori” (Schelling 1856–1861, 1.4, p. 530). The only way we can guarantee that our entire system is true rather than merely hypothetical, Schelling believed, is by ascertaining that its first principle is certain and by deriving all other propositions from this first principle (Schelling 1856–1861, 1.3, p. 277).

In denying that induction can yield the certainty required of propositions and principles in a proper science, Schelling does not mean to imply that induction and experiment have no place in science. As Schelling sees it, and as is clear from his methodology in the 1797 *Ideen zu einer Philosophie der Natur*, the task of experimental science is to discover the “intermediate principles” through which the phenomena and the higher principles of nature are connected (Schelling 1856–1861, 1.3, p. 279). This means that there is room in Schelling’s conception of science for an analytic part in which induction plays a role. What the analytic part does, then, is to discover probable principles whose certainty can subsequently be secured if they can be synthetically ordered in a deductive system and derived from certain a priori principles. The discovery of probable principles is a task for experimental science, whereas the subsequent deduction of these principles from certain a priori principles is a task for speculative physics.

Schelling’s methodological reflections match his scientific procedure in *Ideen zu einer Philosophie der Natur*, his first attempt at a formulation of his program for *Naturphilosophie*. That work starts “from below” rather than “from the top” in that it begins with a discussion of empirical findings from recent science rather than with a discussion of principles (Schelling 1856–1861, 1.2, p. 56). Schelling remarks that the empirical findings discussed in this first part remain uncertain until they are deduced from the principles established in the second part (Schelling 1856–1861, II, p. 4), which starts with a *transcendental argument* that establishes the concept of matter and the highest principles of *Naturphilosophie.* Schelling called this transcendental argument a transcendental exposition of the concept of matter (Schelling 1856–1861, 1.2, p. 214) as well as a “construction” of the concept of matter. By means of this argument, Schelling seeks to show that the concept of matter involves an interplay between an attractive and a repulsive force and that attractive and repulsive force are conditions of the possibility of outer intuition (Schelling 1856–1861, 1.2, p. 216). Having established the highest synthetic a priori principles of natural science in this transcendental manner, Schelling then goes on to present his system of science as an axiomatic deductive system. This is clear from Schelling’s deduction of the axioms of chemistry.

In his deduction of the axioms of chemistry, Schelling proceeds *more geometrico*, an axiomatic presentation he deems necessary for the presentation of science (Schelling 1856–1861, 1.2, pp. 316–317). He first presents the single fundamental dynamical principle of chemistry, which is justified transcendentally. On the basis of this principle, together with several definitions, he deduces the axioms of chemistry. For example, he defines homogeneity as follows: “[t]hose substances are said to be *homogeneous* in which the quantitative relationship of the basic forces is the same” (Schelling 1988, p. 252; Schelling 1856–1861, 1.2, p. 317). The principle and definitions provide the basis for deducing the first axiom, according to which “[n]o chemical process is anything else but an interaction of the basic forces in two bodies” (Schelling 1988, p. 253; Schelling 1856–1861, 1.2, p. 318). On the basis of the definitions and the first axiom, Schelling then deduces the second axiom, which states that “[b]etween *homogeneous* basic substances, *no* chemical process occurs.” This deduction proceeds as follows: (a) The quantitative relationship of the basic forces is more or less the same in homogeneous substances (from the definition of homogeneity). (b) Hence, no change or interaction in these quantitative relationships can occur (direct inference from premise (a)). (c) Hence, there cannot be a chemical process between the substances (from the first axiom) (Schelling 1988, p. 253; Schelling 1856–1861, 1.2, p. 318). This is clearly meant as a deductive demonstration of a *synthetic* proposition from synthetic premises. This supports our interpretation of Schelling as someone who, once he has established the synthetic principles of natural science via transcendental argument, seeks to present an axiomatic system of science.

Importantly, Schelling also believed that his system allowed him to arrive at the principles of organic nature. In the *Erste Entwurf*, he proclaims that his science also aims to explain organic phenomena on the basis of natural forces (Schelling 1856–1861, 1.3, p. 273). But it was mostly the 1798 *Von der Weltseele* that first outlined this project. In that work, Schelling seeks to establish that the opposition between mechanism and organism is false and to demonstrate philosophically that organism explains mechanism and is the condition of mechanism (Schelling 1856–1861, 1.2, pp. 349–350). Schelling thus wants to show that a single principle governs both the organic and the inorganic realm. To this end, he offers an account of life and of how life harmonizes with his Kant-inspired dynamic theory of matter (for Schelling’s concept of life, see Ostaric 2001). In this way, he hoped to bypass Kant’s pessimistic judgement of the possibility of a proper science of life by showing how organic phenomena can be deduced from the metaphysical foundations of natural science, which he, like Kant, believed to involve a theory of matter. This was one of the steps that emboldened him to discard the Kantian restriction of teleology to regulative judgment and offer constitutive principles for the life sciences. According to Richards (2002, p. 294) and Zammito (2018, p. 316), it is this dismissal of Kantian restraint that qualifies Schelling as a major contributor to the development of the science of biology. This is true, but Schelling believed he could move away from Kant because he thought he had found a way to do what Kant thought could not be done: namely, to show how the specific principles governing the organic were grounded in the fundamental principles of natural science which stem from the dynamic theory of matter. Far from dismissing Kantian strictures on proper science, Schelling instead sought to show how the life sciences could satisfy them.

Schelling’s program for *Naturphilosophie* was an attempt to ground all branches of science into a single fundamental principle and to derive from that principle the great variety of natural phenomena. But this implies that both organic and inorganic phenomena can find a home in the same overarching science. As Henrik Steffens already saw, the ultimate outcome of Schelling’s *Naturphilosophie* is that "[i]n such a science all the divisions of natural inquiry into physics, chemistry, physiology, etc. as sciences set apart from one another will fall away, for its purpose would be the unification of all these branches under higher principles" (Steffens 1800, p. 6; quoted and translated in Zammito 2018, pp. 496–497). It seems, then, that although Schelling’s program allows for scientific inquiry into organic phenomena, it leaves little room for an autonomous science of life. In the next section, we will see that this worry motivated Gottfried Reinhold Treviranus.

## The Axiomatic Method in Treviranus’s *Biologie*

One of the main reasons why Gottfried Reinhold Treviranus has received attention from historians of the life sciences is because he authored the *Biologie, oder Philosophie der lebenden Natur für Naturforscher und Aerzte*, the first volume of which appeared in Göttingen in 1802, with the other five volumes appearing in the course of the following 20 years. Because it is one of the first books to mention the term *biology*, in the sense of the “science of life,” on its title page, it is considered a milestone in the emergence of biology as a science. But its exact role in that emergence is more difficult to assess. Joan Steigerwald has noted that Treviranus’s *Biologie* “provides an ambiguous prototype for a science of biology” because Treviranus established neither a new school or discipline nor created an institutional or technical basis for empirical inquiry into life (Steigerwald 2014, p. 105). Nevertheless, several possible contributions to the emergence of biology have been found in Treviranus’s work. Timothy de Jager (1991, pp. 44–45) has suggested that Treviranus paid specific attention to the dynamics of nature and in this way helped pave the way for a more transformist and historical conception of nature. According to Andrea Gambarotto, Treviranus’s work is mostly synthetic, because it brings together various strands of earlier work (2018, p. 93) and follows Schelling in many ways, but goes beyond the latter in presenting the deduction of the “ideal series” of organisms as a “real” and therefore historical sequence of organic forms (Gambarotto 2018, p. 113). Thus, like de Jager, Gambarotto considers the historicization of nature and of life to be the aspect of Treviranus’s thought that contributed to the emergence of biology. Steigerwald focuses more on Treviranus’s interest in the boundaries between living and non-living nature as indicative of his sensitivity to pressing issues in the emerging science of biology, although she argues that his research on this boundary between living and non-living nature may actually have ended up undermining his attempt to strictly demarcate living from non-living nature (2014, p. 107).

In this section, we argue that Treviranus intended to contribute to the establishment of biology by providing a framework that would allow biology to meet the requirements of an autonomous proper science as expressed by the axiomatic ideal of science. That Treviranus meant to make biology into a proper science and to cast it in the form of a system has been recognized. Steigerwald notes that “[i]n the lengthy introduction in the first volume [of his *Biologie*], Treviranus laid claim to scientific study of living organisms, modeled after philosophical conceptions of science as a system of knowledge … and founded on certain principles” (Steigerwald 2014, p. 106). Gambarotto points out that, for Treviranus, “[t]rue science must be based on fundamental principles and deduce all its propositions from them” and that this demand reveals Kant’s and Schelling’s influence (Gambarotto 2018, p. 94). However, apart from these brief and general remarks, these authors do not discuss this aspect of Treviranus’s work in more detail and focus instead on its more historicist and naturalist strands. As a result, the specific logic and methodology Treviranus employs remains unclear. Moreover, although Steigerwald and Gambarotto point towards its Kantian and Schellingian origins, they do not treat Treviranus’s conception of science as a variety of the axiomatic ideal of science, which, as we have shown, remained influential at the beginning of the nineteenth century. Here we show that, although Treviranus did contribute to the naturalist and historicist strands of thought that are crucial to the development of biology as a discipline, he also responded to the need of founding a proper science of biology by trying to cast biology as an axiomatic system. Through our analysis, the logic and methodology employed by Treviranus comes into sharp focus. We argue, moreover, that, although Treviranus followed Schelling’s lead in many instances, he intended to part ways with Schelling on the issue of the autonomy of biology. We show this by discussing Treviranus’s remarks on the concept of a science, his attempt to deliver a Kantian and Schellingian inspired metaphysics of natural science, and his view on the role of empirical inquiry in proper science.

In the preface to *Biologie*, Treviranus motivates his attempt to ground a general science of life by pointing out the sorry state of natural history, which is sitting on a massive wealth of descriptions and observations but lacks a satisfying system with which to consolidate this disparate knowledge (Treviranus 1802–1822, 1, pp. iii–iv). This complaint corresponds to what Wolf Lepenies has called the “growth crisis” of natural history: “For ages, naturalists had been concentrating on the expansion of their knowledge, before they noticed that their means of knowledge no longer sufficed to achieve systematization of knowledge in this accumulation of facts” (Lepenies 1976, p. 62; our translation). The decline of natural history and the emergence of its successor science, biology, stem in part from a need for systematization of merely empirical, merely “historical” knowledge. Treviranus’s point of departure is therefore similar to that of C.F. Wolff: a sense that the life sciences leave us with merely historical (descriptive) and empirical knowledge, but lack, in Christian Wolff’s terminology, truly philosophical knowledge, that is, knowledge of grounds that explains *why* something is the case.

The opening sentences of the preface to the first volume of *Biologie* reveal that Treviranus endorses the axiomatic conception of science:

The value of wealth consists in use, and not in possession. A limited number of propositions deduced from a supreme principle and connected in a consistent whole is more to be treasured, than all the disconnected facts of a pedant. (Treviranus 1802–1822, 1, p. iii; our translation)

Treviranus contrasts the disparate knowledge of natural history with an axiomatic model of science as based on a single supreme principle. He repeats Fichte’s and Schelling’s descriptions of science as a system almost verbatim, stressing their requirement that the system should be grounded in a single fundamental principle. Moreover, Treviranus states that the propositions of the system ought to be deduced from the supreme principle. It is clear, then, that according to Treviranus, a proper science needs to satisfy conditions (3a) and (3b) of the classical model.

Treviranus’s explicit aim in *Biologie* is to elevate the doctrine of living nature to the rank of a proper science (Treviranus 1802–1822, p. 4). That task is less concerned with conducting new research than with synthesizing the results of past research into a single, axiomatic system. Correspondingly, the Introduction to *Biologie* contains a chapter titled “The Fundamental Propositions of Biology.” In this chapter, Treviranus seeks to clarify the concept of “life” on which the realm of the living can be regarded as a distinct domain of objects, analyzes the concept of matter that allows us to determine whether life can be reduced to matter, and determines the fundamental principles, i.e., the axioms of biology.

Treviranus believes the object of biology to be physical life. Unfortunately, Treviranus complains, there is little clarity or agreement on what life means (1802–1822, 1, p. 16). The first task he sets himself is therefore to clarify this concept. Here we see that Treviranus seeks to clarify the fundamental concept of biology in order to precisely delimit the *domain* of biology (condition (1) of the classical model). To this end, he considers several definitions of life and concludes that they are all unsuited to delimiting the object of biology. This leads him to the claim that the mark of mental life is choice (*Willkür)*. Living beings, Treviranus says, have often been associated with mental life because they exhibit an appearance of choice, namely, the ability to counteract outside influences in such a manner that some of their properties will remain the same even if outside circumstances and influences change (1802–1822, 1, pp. 22–23). He illustrates this conception of life with the example of living beings that are capable of maintaining their core temperature even when the ambient temperature changes (1802–1822, 1, p. 156).

Treviranus proceeds to explain why non-living nature does not exhibit this ability (1802–1822, 1, pp. 24–25). He starts from the dynamic conception of matter offered by Kant (Treviranus 1802–1822, 1, p. 25). This shows that Treviranus, like Schelling, explicitly harks back to Kant’s attempt to provide a philosophical foundation for natural science. Moreover, like Schelling, Treviranus parts ways with Kant on some issues, since he rejects the latter’s claim that matter is only subject to two fundamental forces, attraction and repulsion, that are irreducible to one another. Against this claim, Treviranus argues that we need only assume one of these fundamental forces, since matter always exists together with other matter. Take the case where there is only repulsive force. In this case, two material objects would exert upon each other a repulsive force that leads them to distance themselves ever further from one another. In fact, this would happen between any two pieces of matter. However, this scenario only takes place when the two objects are not surrounded by other material objects. If the universe is full of material objects, the repulsive forces between objects balance out: any repulsive force exerted on an object in one direction is balanced out by another repulsive force exerted on it in the other direction. The same would be true in the case where there is only the attractive force. Hence, Treviranus argued, we need to postulate only one fundamental force, and as the rest of his discussion shows, he settled on regarding the repulsive force as primitive (1802–1822, 1, pp. 25–28). In this way, Treviranus believed to be able to arrive at the fundamental synthetic principles of natural science through a priori analysis.

Like Schelling, Treviranus also argued that his revised version of Kant’s dynamical matter theory allows us to ground or deduce both mechanical and chemical phenomena. We present one of his arguments to illustrate this point. The premise of the argument is the proposition that “all original activity taking place in the universe consists of changes in density of materials and in their movements” (Treviranus 1802–1822, 1, p. 44). Treviranus believes that this proposition can be proved from the assumption that matter only involves a repulsive force, which can only change the position of its center in space (a change that appears to us as motion) or the extent of its sphere of activity. Once proved, this proposition would allow us to deduce the further proposition that “all original changes in the universe are partly chemical, partly mechanical, and the former consist either in expansions or contractions,” with an argument that can be schematized as follows (Treviranus 1802–1822, 1, p. 45):

1. All original activity taking place in the universe consists in changes in density of materials and in their movements (Axiom, proved from other axioms and definitions).
2. Decreased density of a material is called expansion (Definition).
3. Increased density of a material is called contraction (Definition).
4. Expansion and contraction are both chemical changes (Definition).
5. The changes of the center of repulsive forces in relative space appear to us as movement (Premise).
6. The changes of the center of repulsive forces in relative space are called mechanical changes (Definition).
7. All original changes in the universe are partly chemical, partly mechanical, and the former consist either in expansions or in contractions (Conclusion).

In this way, Treviranus meant to show which kind of phenomena can be explained on the basis of the fundamental force of matter alone. His methodology matches Schelling’s procedure in the *Ideen*. First, through a priori analysis, Treviranus establishes the fundamental principles of natural science, captured by his own version of the dynamical theory of matter. Then he *deduces* further principles and propositions from these fundamental principles.

Treviranus takes the most important consequence of his picture of physical nature to be that, since matter is everywhere and forces are at work between all of matter, every change in one part of the physical universe will provoke corresponding changes in every other part (1802–1822, 1, p. 28). Remember that Treviranus defined life as the absence of change under changing external influences. In non-living physical nature, this is impossible, since every change brings about a corresponding change, and absence of change requires that external circumstances remain unchanged. As a result, life is impossible on the assumption that only repulsion is at work in nature. Treviranus concludes that life must therefore be due to a force that is irreducible to the repulsive force and that governs all phenomena of life (1802–1822, 1, pp. 37–38).

In this manner, Treviranus argues that physical life is clearly distinct from the rest of physical nature and is subject to a distinct fundamental force that is irreducible to the fundamental force that governs brute matter. It is important to note that this is also a major difference between Treviranus and Schelling, for Treviranus rejects Schelling’s project of deriving the fundamental concepts of matter and of life from a more basic concept and criticizes those who want to deduce the concepts of life and matter, which are concepts of experience, from a more basic principle, which would have to be being itself or God (Treviranus 1802–1822, 3, pp. 545–546). Treviranus specifically rejects the proposal to deduce the fundamental principles of sciences from the absolute, because such a ground does not have the specificity that would allow one to deduce a specific system from it. Treviranus’s criticism of Schelling is important, not just because he wants to resist possibly pantheistic and Spinozist consequences of the latter’s *Naturphilosophie*, but also because he rejects the attempt to reduce biology to a more fundamental science. As we have seen in the previous section, Schelling envisaged *Naturphilosophie* as a system in which all the phenomena of nature, both organic and inorganic, were derived from a single fundamental principle. Treviranus inherited Schelling’s conception of science, but he rejected Schelling’s attempt to ground biology and inorganic physics in a higher principle. In other words, Treviranus insisted on the autonomy of biology as a science with its own proper domain and its own fundamental principles.

After Treviranus has settled on his definition of life, he proceeds to put it to use in the following manner:

First we will establish the possibility of the state of life from those propositions to which the analysis of the concept of matter leads, and from the character of life; from this we will try to deduce and explain the various phenomena and modifications of life, and will continue with these attempts, until we reach a point where we will need the help of experience. (Treviranus 1802–1822, 1, pp. 42–43)

This method is in line with the classical model of axiomatic science. The goal is to first establish, independently of experience and on the basis of the fundamental concepts of life and of matter, a number of general and necessary propositions. Thus, like Schelling, Treviranus provides an a priori analysis that allows him to establish and justify synthetic fundamental principles. These principles need to concern both life and matter, since *Biologie* is specifically concerned with physical life, that is, with living *bodies* (*Körper*). According to Treviranus, the matter of a living body is subject to the same laws as that of lifeless nature (1802–1822, 1, p. 52). But the fundamental force of matter alone is an insufficient ground for the phenomena of physical life. Hence, biology has its own proper concepts and principles, that is, those concerning life and its specific life-force. These are the primitive concepts and axioms of biology. Having established these fundamental concepts and principles (axioms) of biology a priori, Treviranus insists, again like Schelling, that these fundamental concepts and fundamental principles can be used to *deduce and explain* the phenomena and modifications of life. This means that he regards statements about such phenomena as grounded in the fundamental principles. In sum, Treviranus believes that, in this manner, biology can satisfy conditions (2a), (2b), (3a), and (3b) of the classical model and that it is therefore an autonomous science.

Treviranus says that this process of deduction can only continue up to a certain point and that, at some point, we need to start referring to experience. This does not mean that he is no longer following the axiomatic method. He regards the empirical part of biology as an analytic part of the axiomatic method, meant to prepare the way for a synthetic part in which the propositions established through experience are deduced from the fundamental principles of biology (Treviranus 1802, p. 117). This is especially evident from his treatment of the laws of reproduction and growth in volume 3 of the *Biology,* published in 1805, the penultimate section of which is explicitly titled: “Attempt at a deduction of the previous empirical propositions from the fundamental principles of Biology.” There, Treviranus draws mostly on two fundamental propositions of Biology which he deduced in his introduction, namely that.

(i) An individual’s reactions against unequal influences from the outside world can only be equal within certain limits. That is to say, if the outside influences are too strong, the individual can no longer remain indifferent to them and its ability to maintain its uniformity can be impaired. (Treviranus 1802–1822, 3, p. 552)
(ii) Every living individual depends on the totality of the world, which is like a great organism in which everything is connected and cause and effect of each other. (Treviranus 1802–1822, 3, p. 552)

From these two fundamental principles, Treviranus deduces, for example, that living systems form a linear series from the highest to the lowest degree of life, i.e., from the highest to the lowest degree to which a system can maintain uniformity in the face of changing circumstances and influences. However, an organism cannot have a higher degree of life in all respects; if one organism were superior to all others in all respects, Treviranus reasons, it would soon overtake all others and there would be less variety in forms than nature actually exhibits. Hence, to the extent that an organism is more powerful in one respect, it must be less powerful in another (Treviranus 1802–1822, 3, pp. 553–554). This, Treviranus claims, leads to the laws concerning the continuity of living forms which he established from experience in an earlier book, such as “that the size of the brain in comparison with the thickness of the nerves decreases, and the size of the ganglia increases, the further we descend from man to worms [in the series of organization]” (Treviranus 1802–1822, 1, p. 460; our translation). Such laws are specific cases of the more general principle that there must be an inverse relationship between the intensity of functions or capacities in a single organism. In this manner, Treviranus seeks to deduce a variety of laws established through experience from the fundamental propositions he established a priori from the basic concepts of life and matter in the introduction.

Treviranus also accords an important role to experiments in the analytic part of his science. Unlike induction, Treviranus says, experimentation can yield certainty (Treviranus 1802–1822, 1, p. 125). Experiments can therefore be used to confirm mere conjectures. One important example of Treviranus’s use of experimentation to establish more fundamental propositions in biology can be found in the second volume of the *Biologie*, which was published in 1803. There, Treviranus proposes to use experiments to decide on an issue that he was not yet in a position to prove in his introduction, namely, the issue of the relationship between life force and organic matter. He argues that there must be a specific organic matter in nature, basing his reasoning on a review of the experimental literature on infusoria stemming mostly from the mid-eighteenth century (Treviranus 1802–1822, 2, pp. 266–267), such as the experiments conducted by Buffon’s close collaborator John Turberville Needham (1713–1781), the latter’s challenger Lazzaro Spallanzani (1729–1799), the Göttingen anatomist Heinrich August Wrigsberg (1739–1808), the Danish naturalist Otto Frederik Müller (1730–1789), and the Dutch physician Jan Ingenhousz (1730–1799). In addition to reviewing this literature, all of which he took to support the thesis that nature contains specifically organic matter, he conducted experiments of his own as well. Treviranus therefore clearly considered experimentation important, and he can be situated in a tradition of experimental biological research. Our example shows that Treviranus viewed axiomatic biology as an ideal: in biology we are sometimes confronted with empirical propositions that we cannot yet order in an axiomatic system. However, it is also clear from our analysis that Treviranus wanted to take steps to achieve this ideal. Like C.F. Wolff, he wanted to combine experimental research with an axiomatic and deductive presentation of science. As we have shown, the ideal of science that Treviranus endorses is that of experimentally establishing propositions within the analytic part of biology, which are subsequently ordered into a deductive system and derived from the fundamental principles or axioms of biology in the synthetic part of biology.

Treviranus was equally adamant that an empirical approach alone did not suffice and that theory was required in the life sciences. He mentions, for instance, that mere conjectures (*Vermutungen*) are unfit as starting points for further deduction (1802–1822, 2, p. 381). Moreover, Treviranus believes that a variety of issues debated in physiology and medicine are actually undecidable in the absence of clear concepts and higher principles. He complains, for instance, that the concepts of health and sickness are unclear as long as the concept of life is unclear—a concept that needs to be settled upon by the science of biology. He criticizes “physicians” for not “founding [*begründen*] a philosophical theory of their art” and for having proposed “explanations of health and illness, that are not drawn from higher antecedents [Vordersätze], and are therefore inadequate” (1802–1822, 1, p. 9). This shows that Treviranus had in mind a clear hierarchical relationship between the life sciences. On his picture, the fundamental principles and the concepts of the life sciences stem from biology. The other life sciences, like medicine, can then be made more scientific to the extent that they can derive their principles from biology. Ideally, Treviranus suggests, all propositions making up a science would therefore be “as true and indubitable as any part of mathematics” (1802–1822, 1, p. 124). But Treviranus also admits that this an ideal and that, in the meantime, medical practice must partly rely on hypotheses and conjectures, while we strive to acquire more and more certain knowledge.

In conclusion, *Biologie* is an attempt to establish an autonomous proper science of life. The conception of proper science used here is clearly indebted to Kant’s and Schelling’s ideas and is a variety of the classical model of axiomatic science. Biology comprises an analytic and a synthetic part. The analytic part seeks to establish propositions on the basis of experience and experiment. The synthetic part seeks to deduce these propositions from fundamental principles. This is all in line with Schelling’s prescriptions from the *Entwurf*. But an important specific feature of Treviranus’s approach is that he does not endorse Schelling’s attempt to derive the principles of living and non-living nature alike from a single higher principle or science, and denies that the fundamental principle of life can be derived from the fundamental principle of non-living nature. This reveals important parallels between Treviranus’s endeavor and that of C.F. Wolff. Like Wolff, Treviranus sought to formulate the fundamental concepts and axioms of the life sciences. Moreover, both Wolff and Treviranus saw the relationship between the life sciences as hierarchical. Just as Wolff believed that his theory of generation could provide the foundations from which the propositions of anatomy and physiology could be explained, Treviranus believed his biology could be the fundamental science that grounds natural history and medicine. Finally, both Wolff and Treviranus took their fundamental principles to concern a specific domain of objects and phenomena, namely, organic nature and living nature, respectively, thus allowing for the demarcation of the domain of biology. On the basis of our analysis of Wolff and Treviranus, we can therefore conclude that the attempt to specify the foundations of the life sciences and the attempt to treat the life sciences axiomatically were deemed important in the project of establishing biology as an autonomous and proper science.

## Conclusion

In this paper, we have argued that Caspar Friedrich Wolff and Gottfried Reinhold Treviranus adopted an axiomatic ideal of science and that this ideal of science informed the early nineteenth century attempt to ground biology as an autonomous science. C.F. Wolff adopted this axiomatic ideal of science from Christian Wolff, whereas Treviranus adopted it from the conception of science articulated by Fichte and Schelling. What is distinctive of biology as a proper axiomatic and autonomous science is that it has a specific domain of investigation that delimits it from other sciences and that it has fundamental concepts and principles (axioms) that are specific to biology and are not reducible to the concepts and principles of other sciences. This distinguishes the conception of life science adopted by C.F. Wolff and Treviranus from the conception of life science adopted by Christian Wolff. The latter viewed all sciences as axiomatically structured, but treated the life sciences as parts of physics that do not have fundamental concepts and principles of their own. It is also distinct from Schelling’s conception of life science, on which it ought to be grounded in a higher principle that also grounds physical science. The ideal of biology as an axiomatic science with proper biological fundamental concepts and principles thus played a role in the emergence of biology as a special science.

## Footnotes

1 Unfortunately, as Joan Steigerwald’s new monograph on the emergence of German biology, *Experimenting at the Boundaries of Life,* reached us only during the penultimate stages of the publication process, we have not been able to draw on it in the research we present here. Here, however, we provide some general comments on how this book, which we are still studying, relates to the research presented in this paper. Of the “series of challenges to the larger histories of biology and histories of philosophy that we have been telling ourselves” (Steigerwald 2019, p. 40) Steigerwald poses in this book, the one that strikes us as most directly relevant to our present thesis and argument is her contention that “German understandings of organic vitality were informed more concretely and specifically through experimental inquiries than through theoretical systems” (Steigerwald 2019, p. 6). In her book, Steigerwald stresses the role of experimental demonstrations involving “sophisticated modes of reasoning as well as practice,” which “not only made evident new modes of organic vitality, they also demonstrated their character” (Steigerwald 2019, pp. 7, 8). In this way, she argues that, far from being mostly driven by simple empiricism or speculative theory, the development of biology in the eighteenth century, and especially in German romanticism, was driven by sophisticated experimental practices. In this sense, Steigerwald’s work may seem to point in the very opposite direction from what we claim here, namely that what is driving the emergence of biology is not so much a traditional “justificationist” (to borrow a term from Lakatos 1978, p. 121) theory of what a completed system of science ought to be like (our classical model), as an excitingly modern view of what the practice of science as a material process is actually like. But it seems to us that this opposition between the two images of science, or between our analysis and the analyses offered by Steigerwald, may very well be a false one. On our reading, the historical actors did not believe that adherence to the classical model ruled out respect for or engagement in real experimental practice. On the contrary, we constantly stress that, for most of these authors, experimentation plays a major, perhaps even an indispensable role in science. But in interpreting the results of experiments, the historical actors were attentive to the role those results should play in the body of a scientific system. Some of them were explicitly interested in finding experimental demonstrations for principles that could serve as the foundations of an autonomous science, and as explanations of the variety of organic phenomena, even though these experimental demonstrations may also have had other—intended or unintended—functions. Hence, we may be pointing towards different contributing factors in the emergence of biology rather than to conflicting explanations of that emergence. When we argue here against those who stress the empiricist or experimentalist dimensions of the emergence of biology, this is mostly because we want to resist the idea that these dimensions detract from the role that the classical model, in its many variants, could have played in that development.

2 We use the terms *analytic method* and *synthetic method,* following de Jong (2010, pp. 239–240), in their directional sense. As de Jong explains, the analytic method proceeds in the direction of the principles of a science, whereas the synthetic method proceeds in the opposite direction. More specifically, analysis proceeds from the consequences to the principles, from the effects to the causes, from the whole to the parts, and from the more particular to the more universal, whereas synthesis proceeds from the principles to the consequences, from the causes to the effects, from the parts to the whole, and from the more universal to the more particular. Christian Wolff, who will be the focus here, takes analysis to involve inductive steps to establish propositions (which we discuss in another paper) and also took analysis to involve deductive inferences, which we discuss below (the syllogistic inference of the proposition that “air has an expansive force,” discussed below, is an analytic *deductive* inference). Synthesis, for Wolff, is often construed as a deduction proceeding from the principles.

3 The following series of syllogisms is taken from the account of Wolff’s physics given in van den Berg 2014, pp. 31–33.

4 The importance that Wolff assigns to experience in science leads Vanzo (2015) to classify Wolff as an experimental philosopher. However, Vanzo also acknowledges that Wolff adopts a mathematical demonstrative method.

5 Wolff distinguished a posteriori and a priori reasoning as follows: “In establishing truth with our own abilities, we either make use of mere sense, or, by reasoning from what is known, we elicit what is not yet known. In the former case we say that the truth is established a posteriori, whereas in the latter case we say that it is established a priori” (C. Wolff [1740] 1983, II, p. 481; our translation). Here, Wolff identifies a priori knowledge with knowledge established by inference from prior knowledge and not with knowledge established independently of experience. He therefore allows the use of premises that are established a posteriori in a priori reasoning. This feature of Wolff’s use of the term “a priori” has been noted in the literature. Lewis White Beck argues that it is this identification of “a priori” with “derived through a valid syllogism” that made the Wolffians believe that “they could get all the advantages of a rationalistic theory of knowledge while still maintaining an empiricistic basis” (Beck 1996, p. 265). Similarly, Vanzo (2015, pp. 242–244) states that “Wolff’s notion of the a priori is so weak that early modern experimental philosophers could have easily admitted the existence of a priori truths in Wolff’s sense,“ i.e., in the sense of “derived through valid syllogism,” and Favaretti Camposampiero (2016, p. 365) calls Wolff’s notion of the a priori “mild” and “procedural” and notes that it is all but synonymous with “inferential.” Given what he meant by “a priori,” there are no reasons to assume that Wolff’s respect for empirical or experimental inquiry would have weakened his commitment to the demonstrative ideal of science.

6 In this section, we draw on and closely follow the account of Wolff given in van den Berg (2014, pp. 66–69). For a different discussion of Wolff’s view on the life sciences, and in particular his views on medicine (which we cannot treat here), see the illuminating Favaretti Camposampiero (2016). Like van den Berg, Favaretti Camposampiero stresses the importance of a priori concepts and demonstration in Wolff’s conception of the life sciences against the overemphasis of Wolff’s experimental bend in the literature.

7 Favaretti Camposampiero (2016, pp. 367–368) has argued that Wolff’s explicit subsumption of both anatomy and physiology under physics is difficult to reconcile with other aspects of his work, specifically with the fact that he also admits that there is a part of natural philosophy concerned with teleology, and therefore includes principles that are not exclusively physical, but stem from rational theology. Moreover, Favaretti Camposampiero notes that Wolff sometimes characterizes physiology as a science concerned with ends, and therefore not fully reducible to physics (which deals with efficient causation exclusively). These complications may mean that there was room for an aspect of the study of life that does not reduce to physics according to Wolff, or that he may have been unclear on how teleology fit into the system of science. Nonetheless, Wolff does not seem to have considered the life sciences as autonomous in the manner of interest to us here. Whether Wolff recognized some science of life to have autonomous principles or not, he did not consider anatomy and physiology insofar as they deal with efficient causation to be autonomous, and did not subsume the latter sciences under the life science that could be considered autonomous. Moreover, Wolff does not seem to have considered the principle of teleology to be specific to the domain of the living. As we show below, C.F. Wolff did explicitly seek a specific principle for living nature and to ground the life sciences jointly in this principle.

8 On fundamental propositions or judgments, see, for example, C. Wolff ([1754] 1978, p. 161); Meier (1752, pp. 514–515); Lambert (1771, I, pp. 61–62); Lambert (1764, I, pp. 95–96, 571); Kant (1998, KrV, B14).

9 On Lambert, see Heis (2014, pp. 616–617); Dunlop (2009, pp. 48–52); Laywine (2010, pp. 114–121).

10 On how, in Kant, a priori principles ground empirical propositions such as the law of gravitation, see Friedman (1992a, Chaps. 3–4, and 1992b). See also Zammito (2017, p. 473), who, referring to the classical model, takes the Aristotelian ideal of scientia to animate Kant’s sense of proper science. Plaass (1994, pp. 235–236) and Sturm (2009, p. 153), who partly follows Plaass, argue that Kant did not accept an axiomatic conception of science. According to Plaass, it is difficult to see how empirical principles can fulfill a function in axiomatic sciences. Since physics is based on empirical principles, it cannot be an axiomatic science. However, as we have shown in our discussion of Christian Wolff, Wolff allowed for the possibility that principles and propositions of a science are established empirically (and are thus empirical principles), while also arguing that once these principles or propositions are established, we must order sciences axiomatically. We submit that Kant adopted a similar view. Hence, one can acknowledge the role of experience and empirical principles in science while also adopting an axiomatic conception of science.

11 Note that this fact does not yet conclusively establish that Erxleben (or other authors who adopted the same position) did not accept the axiomatic ideal, for Erxleben may have thought, like Kant, that probable hypotheses can be *transformed* into certainties. In order to answer this question, we require a thorough and comprehensive analysis of the notion of *hypothesis* in early modern physics, which we cannot undertake here.

12 Insightful discussions of Wolff’s theories have been provided by Duchesneau (1982), Gaissinovitch (1990), Mocek (1995), Müller (1984), Roe (1979, 1981), Jahn (1998–1999), Detlefsen (2006), Goy (2014), and Zammito (2018, 2019). Since we assess the impact of the ideal of axiomatic science on Wolff’s biology, we will mainly focus our attention on the interpretations of Roe (1979), Detlefsen (2006), and Zammito (2018), which also deal with this topic.

13 Detlefsen cites Duchesneau, who ascribes to Wolff a strategy which involves deriving a probability and arguing from analogy. However, the passage from Wolff on which Duchesneau bases his view is consistent with our reading. In this single passage, Wolff (C.F. Wolff [1759] 1999, pp. 137–140) states that he could offer a demonstration, i.e., a deductive argument, such that the argument would not rely on analogy, and adds that he makes his theory “highly probable” here, noting that some readers prefer probability to demonstrations. Wolff therefore suggests that in this specific instance his strategy is for the sake of his readers rather than due to his own methodological preferences. The fact that Wolff distinguishes in this way between proper demonstrations, of which he offers many throughout his work, and the merely probable arguments he is supplying here for his readers’ sake, harmonizes with our reading.

14 We refer to both the page number of the English translation and the standard volume and page number according to the Fichte Gesamtausgabe der Bayerischen Akademie der Wissenschaften (GA).

15 See, for instance, Pearce Williams (1973, p. 17), Esposito (1977, p. 49), Friedman (2006, p. 58; 2013, pp. 76–77), and Zammito (2017, pp. 302–303).
